# The Effects of Drug Addiction and Detoxification on the Human Oral Microbiota

**DOI:** 10.1128/spectrum.03961-22

**Published:** 2023-02-01

**Authors:** Jun Zhang, Wenli Liu, Linyu Shi, Xu Liu, Mengchun Wang, Wanting Li, Daijing Yu, Yaya Wang, Jingjing Zhang, Keming Yun, Jiangwei Yan

**Affiliations:** a Shanxi Medical University, Taiyuan, People's Republic of China; b Beijing Center for Physical and Chemical Analysis, Beijing, People's Republic of China; Ohio State University

**Keywords:** drug addiction, detoxification, heroin, methylamphetamine, oral microbiota

## Abstract

Drug addiction can powerfully and chronically damage human health. Detoxification contributes to health recovery of the body. It is well established that drug abuse is associated with poor oral health in terms of dental caries and periodontal diseases. We supposed that drug addiction and detoxification might have significant effects on the oral microbiota. To test the hypothesis, we assessed the effects of drug (heroin and methylamphetamine) addiction/detoxification on the oral microbiota based on 16S rRNA gene sequencing by an observational investigation, including 495 saliva samples from participants. The oral microbial compositions differed between non-users, current and former drug users. Lower alpha diversities were observed in current drug users, with no significant differences between non-users and former drug users. Heroin and METH addiction can cause consistent variations in several specific phyla, such as the enrichment of Acidobacteria and depletion of Proteobacteria and Tenericutes. Current drug users had significantly lower relative abundances of Neisseria subflava and Haemophilus parainfluenzae compared to non-users and former drug users. The result of random forest prediction model suggested that the oral microbiota has a powerful classification potential for distinguishing current drug users from non-users and former drug users. A cooccurrence network analysis showed that current drug users had more complex oral microbial networks and lower functional modularity. Overall, our study suggested that drug addiction may damage the balance of the oral microbiota. These results may have benefits for further understanding the effects of addiction-related oral microbiota on the health of drug users and promoting the microbiota to serve as a potential tool for accurate forensic identification.

**IMPORTANCE** Drug addiction has serious negative consequences for human health and public security. The evidence indicates that drug abuse can cause poor oral health. In the current study, we observed that drug addiction caused oral microbial dysbiosis. Detoxication have positive effects on the recovery of oral microbial community structures to some extent. Understanding the effects of drug addiction and detoxification on oral microbial communities will promote a more rational approach for recovering the oral function and health of drug users. Furthermore, specific microbial species might be considered biomarkers that could provide information regarding drug abuse status for saliva left at crime scenes. To the best of our knowledge, this is the first report on the role of the oral microbiota in drug addiction and detoxification. Our findings give new clues to understand the association between drug addiction and oral health.

## INTRODUCTION

The human oral cavity is colonized by over 700 different bacterial species and other microorganisms (1). These microbes are involved in diverse functions and play a crucial role in human health (2). Previous studies have shown that oral microbiome dysbiosis is not only related to oral diseases (3–5) but is also potentially related to systemic diseases (6–9). Hence, the oral microbiome has been considered to provide essential information for the understanding of oral health and systemic diseases.

The evidence indicates that cigarette smoking (10) and alcohol consumption (11) could alter the oral microbiota and potentially lead to increased colonization by pathogenic bacteria that are associated with smoking-related and alcohol-related diseases, respectively. Drug addiction can powerfully and chronically weaken human health (12). In particular, drug abuse can affect adaptive immunity (13). The characteristics of the adaptive immune system are important determinants for interactions between the microbiome and hosts (14). On the other hand, drug abuse can cause poor oral health in terms of dental caries and periodontal diseases (15). It has been well established that oral microbial community structures vary between healthy host populations and populations with dental caries or periodontal disease (3–5). Furthermore, drug abuse can change the environment of the saliva, such as the pH levels and salivary flow rates (16) and in general, drug abusers have poor lifestyle habits and oral cleanliness (17). Based on the findings, we hypothesized that drug addiction could change the oral microbial community structure.

Detoxification is the process of disengaging a person from drug abuse in a safe and effective manner and involves the recovery of bodily health (18). If the hypothesis that drug addiction could change the oral microbial community structure is true, detoxification might have positive effects on the recovery of the oral microbial community structure to some extent. There is another possibility that detoxification could increase the differences in oral microbial community structures between drug users and non-users. This possibility may be attributed to the high sugar consumption during the process of detoxification, especially for oral methadone solutions that are used to manage heroin withdrawal (19). However, these are merely our speculations, and the effects of drug addiction and detoxification on the oral microbiota are poorly known.

Understanding the effects of drug addiction and detoxification on oral microbial communities will promote a more rational approach for recovering the oral function of drug users. On the other hand, specific microbial species might be considered biomarkers that could provide information regarding drug abuse status and for saliva left at crime scenes. This is helpful for finding investigation clues, shrinking investigation scopes, clearing people of criminal suspicion or identifying criminal suspects.

**FIG 1:**
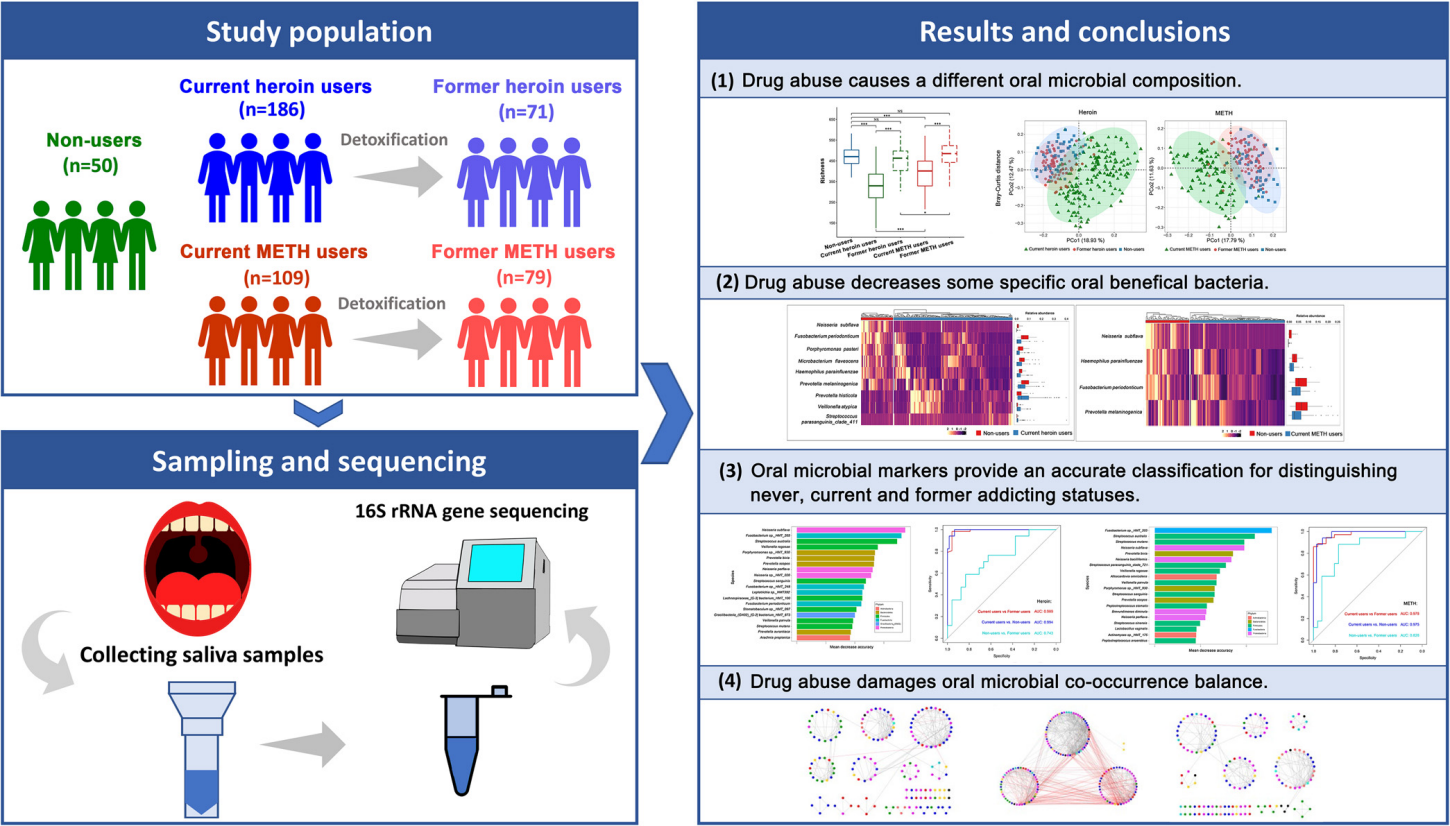
Graphical abstract. Drug addiction may change the human oral microbial composition and damage the balance of the oral health microbiota. These results may have benefits for further understanding the effects of addiction-related oral microbiota on the health of drug users and promoting the microbiota to serve as a potential tool for accurate forensic identification.

Heroin and methamphetamine (METH) are commonly abused drugs that pose a significant economic burden (13, 20). We supposed that drug addiction and detoxification might have significant effects on the oral microbiota. To test the hypothesis, we assessed the microbial community compositions and taxon abundances of 495 oral wash samples, including those obtained from heroin and METH drug user, before and after detoxification by the use of bacterial 16S rRNA gene sequencing (Fig. 1). To the best of our knowledge, we present the first available evidence on the effects of these two drugs on the oral microbial ecology.

## RESULTS

### The differences in oral microbial diversities across addiction status categories.

A total of 38,248,051 high-quality (above Q20) sequences were obtained. Each sample contained 77,269 sequences. Then, 1,711 OTUs were identified as bacteria from our salivary samples. The standard sampling size calculations (Fig. S1) showed that our study had enough sampling sizes to get adequate statistical power (80%).

**FIG 2:**
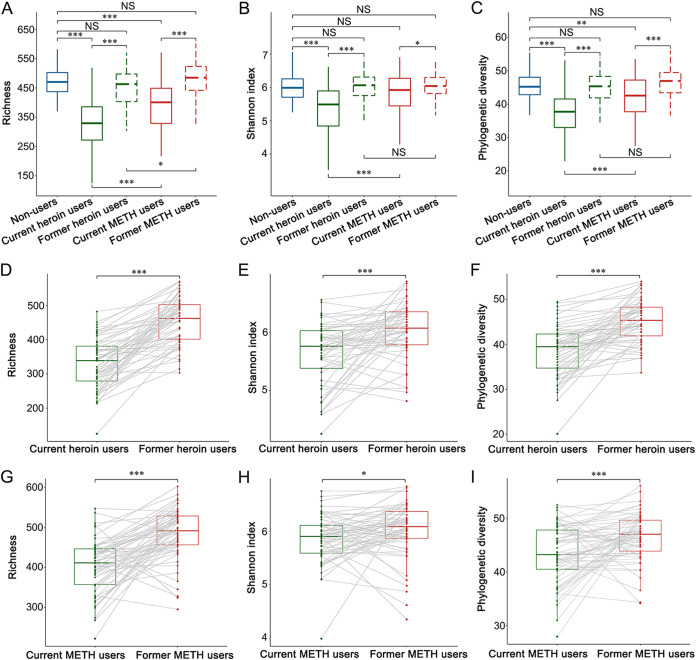
Boxplots showing species richness (A, D, and G), Shannon index (B, E, and H), and phylogenetic diversity (C, F, and I) according to drug addiction status. The statistical differences were analyzed using Wilcoxon tests. A–C were plotted based on the alpha diversities of all samples. Overall, drug addiction decreased the alpha diversities of the oral bacterial communities, and detoxification increased those of drug users. D-I were plotted based on the oral alpha diversities of drug users (heroin: D-F, METH: G-I) who have samples, including both before and after detoxification. The statistical differences were analyzed using pairwise Wilcoxon tests. The results of pairwise statistical tests were consistent with those of all samples. ***, *P* < 0.001; **, *P* < 0.01; and *, *P* < 0.05.

The alpha diversities of the oral bacterial communities decreased caused by drug addiction and increased after drug detoxification (Fig. 2A to C). Specifically, all three alpha-diversity indices of current heroin users were significantly lower than those of non-users and former heroin users. The observed OTUs and PDs of current METH users were significantly lower than those of non-users. All three indices increased significantly after detoxification for METH users. The alpha diversities of former drug users were very close to those of non-users. In addition, heroin had a greater effect on oral microbial alpha diversities compared to METH, all three alpha-diversity indices were significantly higher in current METH user than in current heroin users. After detoxification, only the richness was still significantly higher in METH users than in heroin users. The pairwise tests were also performed for current and former drug users and obtained similar results with those of all samples (Fig. 2D to I).

**FIG 3:**
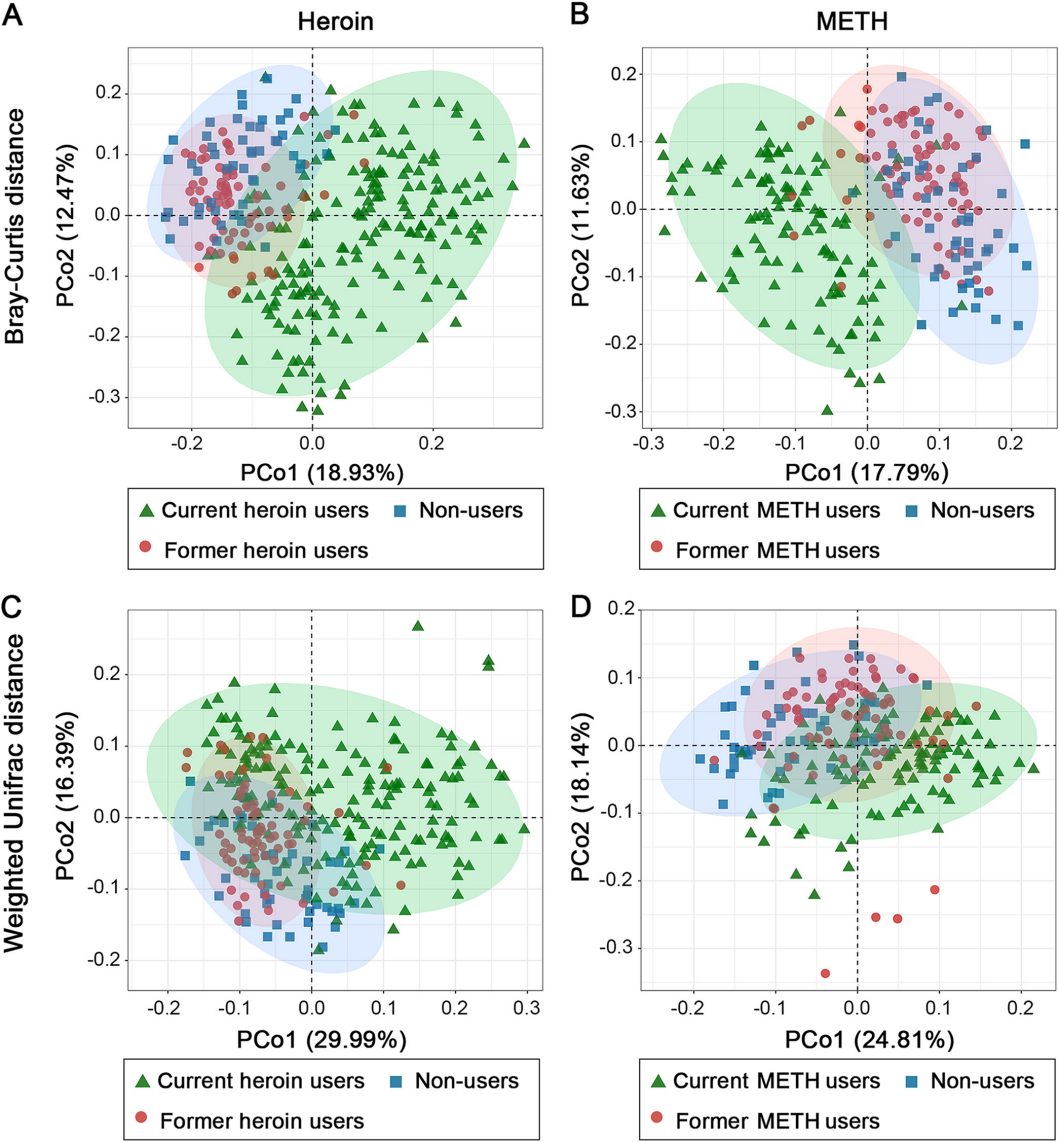
Principal coordinate analysis (PCoA) based on the Bray–Curtis (A and B) and weighted UniFrac (C and D) distances showing the contribution of drug (A and C for heroin; B and D for METH) addicting statuses (e.g., never, current or former) to shaping the compositions of oral microbial communities. The results show that the distribution of current drug users separated from that of non-users, former drug users located between current drug users and non-users.

**FIG 4:**
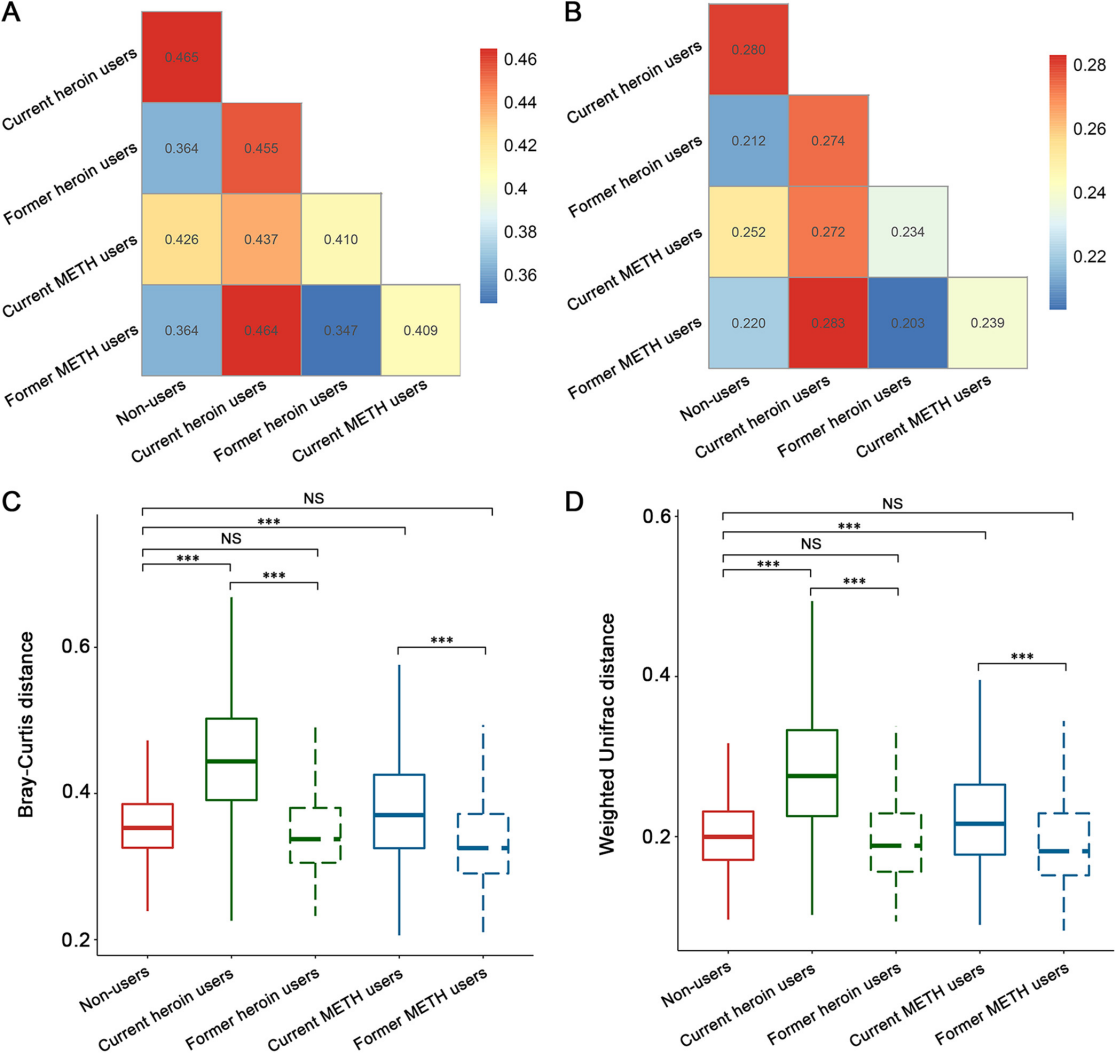
A comparison of the between (A and B) and within (C and D) group distances (A and C for the Bray–Curtis distances, B and D for the Weighted UniFrac distances) for all addiction categories indicated that non-users and former drug users were more alike. The microbial communities of former drug users are more similar to those of non-users than to those of current drug users (A and B). The oral microbial communities of current drug users are more heterogeneous than those of non-users and former drug users (C and D).

The results of PERMANOVA showed that the overall oral microbial compositions significantly (*P* < 0.001) differed across the non-users, current and former drug users after controlling other factors (Tables S1 and S2). The tests only including pairwise data also showed that current and former drug users had significant differences in oral microbial community structure (Table S3). PCoA was performed to confirm the PERMANOVA results with a visualization (Fig. 3 and Fig. S2). Furthermore, the microbial communities of former drug users were more similar to those of non-users than to those of current drug users (Fig. 3 and Fig. 4A and B). In addition, the oral microbiome compositions of current heroin users were significantly (*P* < 0.05) different from those of current METH users, whereas no significant differences were observed between former heroin users and former METH users (Fig. S3, Tables S1 and S2). We further compared the within-group distances for all addiction categories, and the results showed that the oral microbial communities of current drug users were more heterogeneous than those of non-users and former drug users (Fig. 4C and D).

Additionally, the age of drug users had a significant influence on the oral microbial communities of former drug users but had no significant influence on those of current drug users (Table S4). The frequency of drug addiction had little influence on the oral microbial communities of drug users (Table S5).

### Differential microbial taxa to distinguish different categories of addiction status.

At the phylum level (Fig. S4), Bacteroidetes was the dominant taxon, which was followed by Firmicutes and Proteobacteria. Acidobacteria, Actinobacteria, Bacteroidetes, and Firmicutes had higher relative abundances, while Elusimicrobia, Epsilonbacteraeota, Fusobacteria, Patescibacteria, Proteobacteria, and Tenericutes had relatively lower abundances in current heroin users than in non-users. These taxa of former heroin users returned to higher relative abundances that were similar to those of non-users. In addition, lower relative abundances of Acidobacteria, Patescibacteria, Planctomycetes, and Synergistetes, as well as higher relative abundances of Proteobacteria and Tenericutes, were observed in non-users and in former METH users than in current METH users.

**FIG 5:**
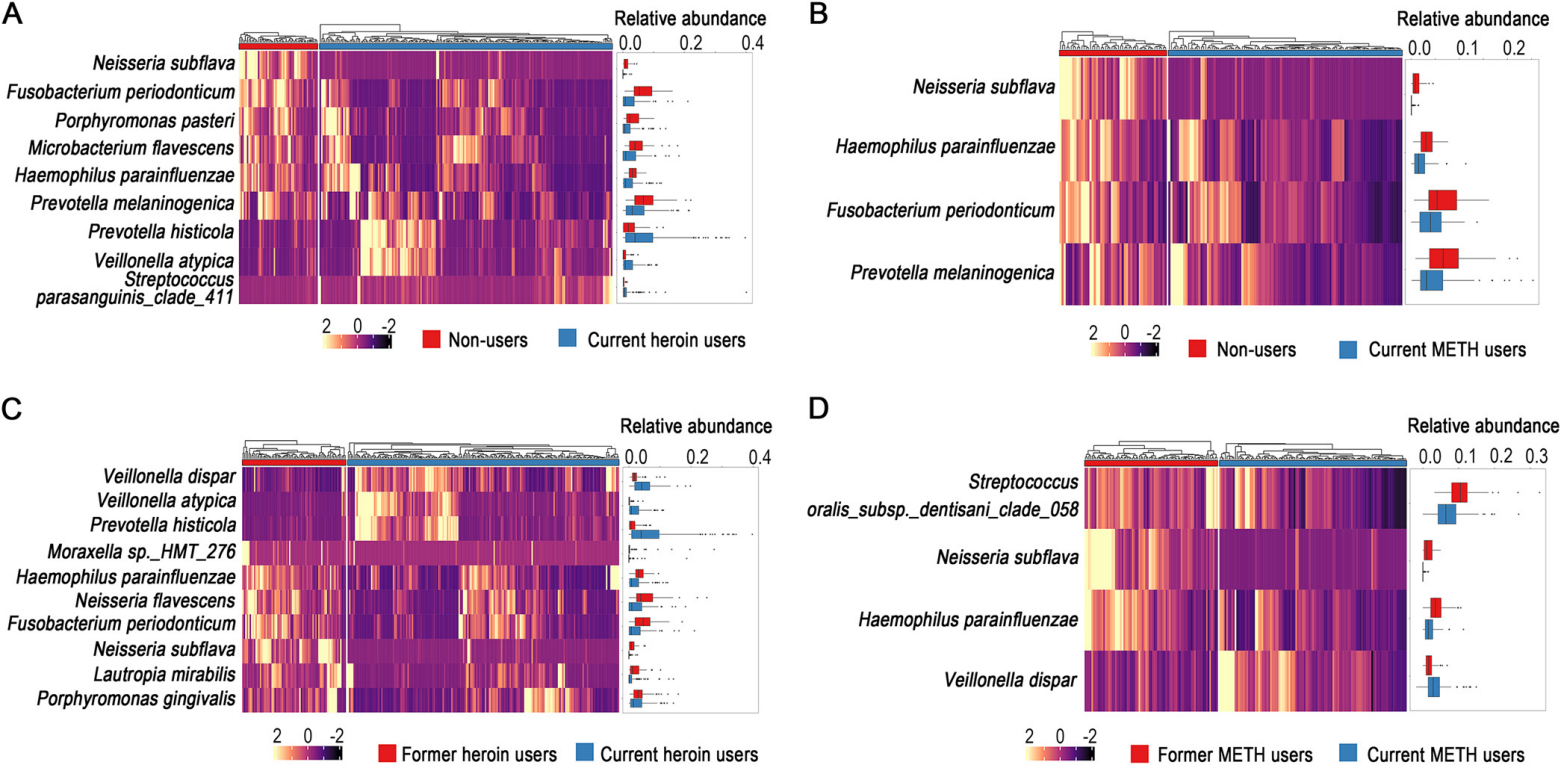
The heatmaps (the values were Z-score transformed) and boxplots show that the species whose absolute difference in relative abundance is significantly (Wilcoxon test, *P* < 0.05) greater than 0.01 when comparing different drug addiction categories. Current drug users had significantly lower relative abundances of Neisseria subflava and Haemophilus parainfluenzae comparing to non-users and former drug users, no matter for heroin users (A and C) or METH users (B and D).

The species whose absolute differences in relative abundances were significantly greater than 0.01 are shown in Fig. 5. There were six and seven species with significantly lower relative abundances in current heroin users compared to non-users and former heroin users, respectively. Among these decreased species, Neisseria subflava, Fusobacterium periodonticum, and Haemophilus parainfluenzae were shared in the comparisons. There were three species with significantly higher relative abundances in current heroin users compared to non-users and former heroin users, respectively. Veillonella atypica and Prevotella histicola were shared in the two comparisons (Fig. 5A and C). For METH users, current users had significantly lower relative abundances of *N. subflava*, *H. parainfluenzae*, F. periodonticum, and *P. melaninogenica* than non-users. The decreases of *N. subflava* and *H. parainfluenzae* were also observed in the comparison of current versus former METH users. Only *V. dispar* had a significantly higher relative abundance in current METH users than in former METH users (Fig. 5B and D).

**FIG 6:**
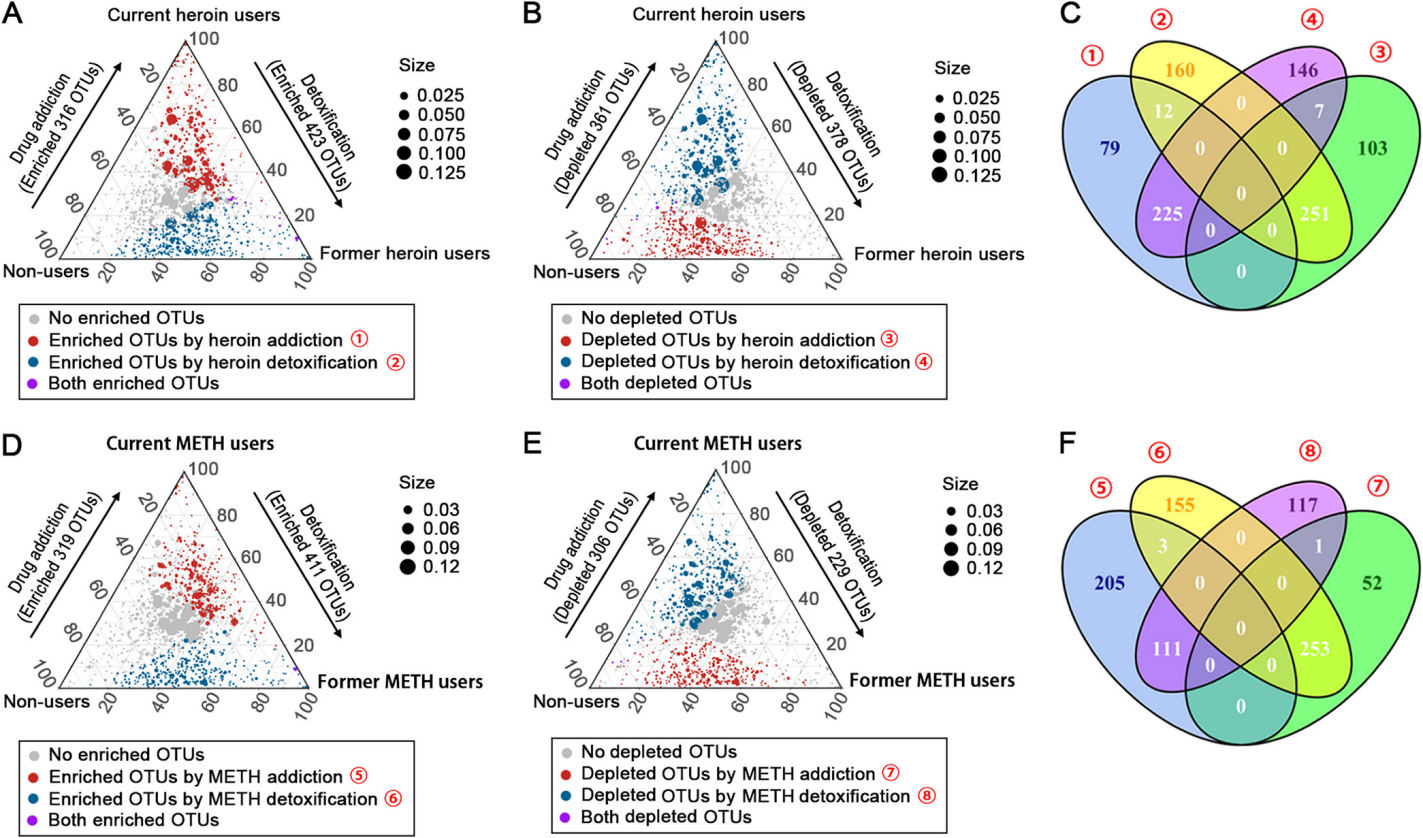
Ternary plots (A, B, D, and E) and Venn diagrams (C and F) depicting the number of OTUs enriched (A and D) and depleted (B and E) caused by heroin (A, B, and C) and METH (D, E, and F) addiction/detoxification. In the ternary plots, each circle depicts one individual OTU. The circle sizes reflect the relative abundances.

We then performed a differential OTU abundance analysis based on the raw abundances by fitting a negative binomial generalized linear model (Fig. 6). Overall, heroin abuse depleted more OTUs and enriched less OTUs compared to METH abuse. For heroin users, 316 OTUs were significantly enriched, and 361 OTUs were significantly depleted in current users compared to non-users, 225 of these enriched OTUs significantly decreased, and 251 of these depleted OTUs significantly increased after detoxification (Fig. 6A, B and C). For METH users, 319 OTUs were significantly enriched, 306 OTUs were significantly depleted in current users compared with non-users, 111 of these enriched OTUs significantly decreased, and 253 of these depleted OTUs significantly increased after detoxification (Fig. 6D, E and F). These results suggested that a significant percentage of changed OTUs due to addiction can be recovered by drug detoxification. In addition, more than half of the changed OTUs caused by heroin and METH were consistent. Current heroin and METH users shared 208 enriched OTUs and 231 depleted OTUs compared with non-users (Fig. S5A), former heroin and METH users shared 284 enriched OTUs and 148 depleted OTUs compared with current users of the corresponding drug (Fig. S5B).

**FIG 7:**
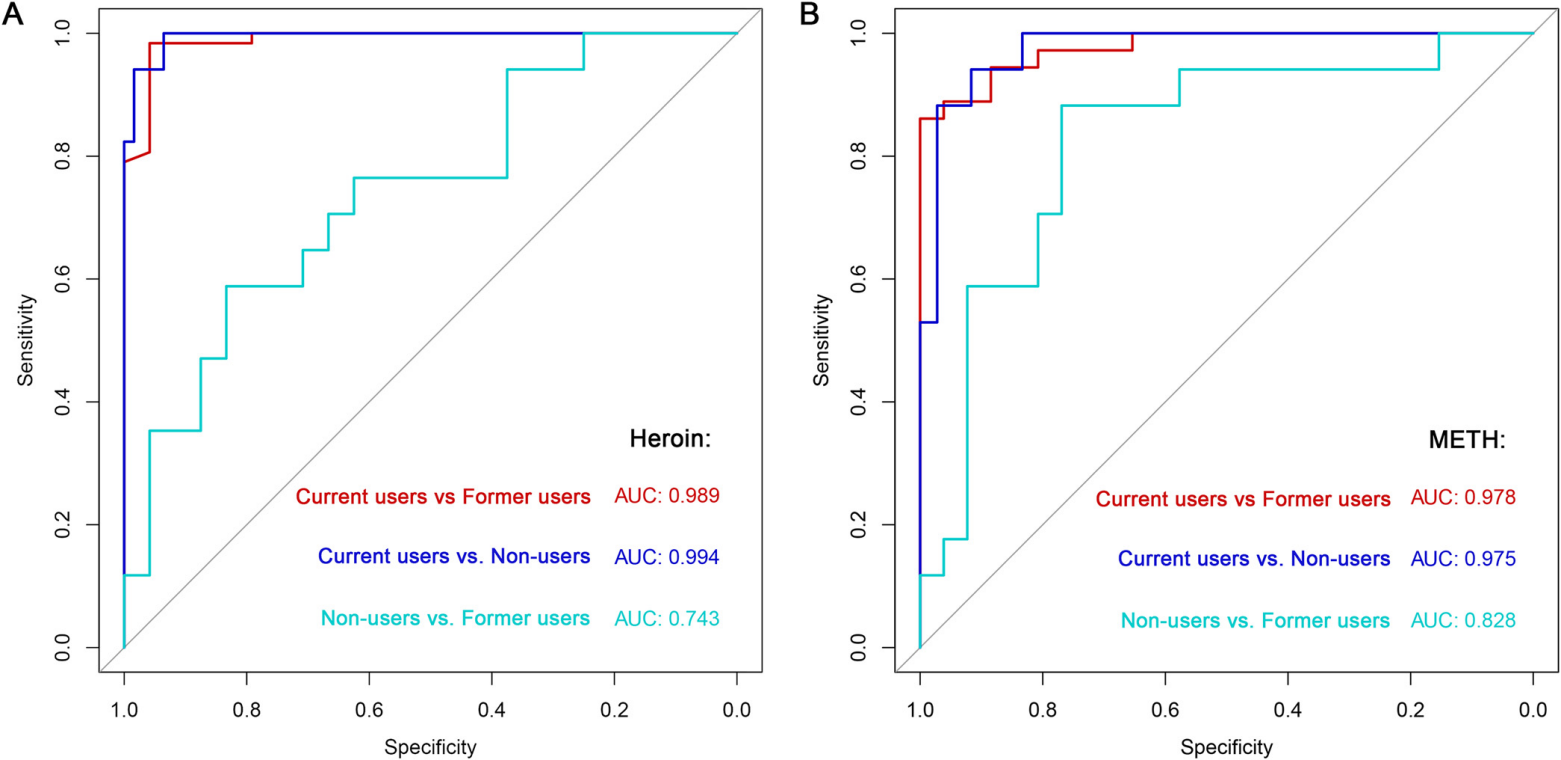
ROC curves depicting the classification performance of the drug (A for heroin and B for METH) addiction categories by using the relative abundances of species based on random forest models. The random forest classifiers provide extremely high prediction accuracies (AUC values range from 0.975 to 0.994) for distinguishing current drug users versus non-users or former drug users.

The results of random forest model showed that 22 (Fig. S6A), 45 (Fig. S7A) and 26 (Fig. S8A) species were selected as the optimal markers to distinguish non-users versus current heroin users, current versus former heroin users, and non-users versus former heroin users, and ROC curves were obtained with AUCs = 0.994 (95% CI,0.984 to 1.000), 0.989 (95% CI,0.971 to 1.000) and 0.743 (95% CI,0.587 to 0.898), respectively (Fig. 7A). Meanwhile, 31 (Fig. S9A), 231 (Fig. S10A) and 64 (Fig. S11A) species were selected as the optimal markers to distinguish non-users versus current METH users, current versus former METH users, and non-users versus former METH users, obtained ROC curves with AUC = 0.975 (95% CI,0.942 to 1.000), 0.978 (95% CI,0.950 to 1.000) and 0.828 (95% CI,0.695 to 0.961), respectively (Fig. 7B). These results suggested that the oral microbial communities could provide a powerful diagnostic potential for distinguishing never, former and current addicting statuses.

Among the selected optimal markers, besides *N. subflava* and *H. parainfluenzae* mentioned above, some species in genus of Streptococcus such as *S. australis*, S. mutans, and S. sanguinis were very important in the classifiers when distinguishing current drug users from non-users or former drug users. Most species had lower relative abundances in current drug users compared to non-users or former drug users at the top 20 important biomarker taxa. All of the taxa had higher relative abundances in non-users than in current heroin users (Fig. S6), and only 2 taxa (Brevundimonas diminuta and Peptostreptococcus stomatis) had lower relative abundances in non-users than in current METH users among the top 20 important biomarkers (Fig. S9). A large portion of these depleted taxa caused by drug addiction were also observed in the comparisons between former drug users and current drug users (Fig. S7 and S10). Furthermore, *B. diminuta* as one of the most important taxa in the classifiers had a higher relative abundance in current drug users (Fig. S6-S11).

The oral microbial communities were also able to distinguish heroin and METH users (Fig. S12). Especially for current users, the AUC values of the ROC curves reached at 0.871 (95% CI,0.798 to 0.944), and 93 species were selected as the optimal markers (Fig. S13). We obtained a powerless diagnostic potential for distinguishing former heroin and METH users, and the AUC values of the ROC curves were 0.747 (95% CI,0.611 to 0.883) when 26 species were selected as the optimal markers (Fig. S14).

### Potential interactions among the oral microbes of different addiction status categories.

**FIG 8:**
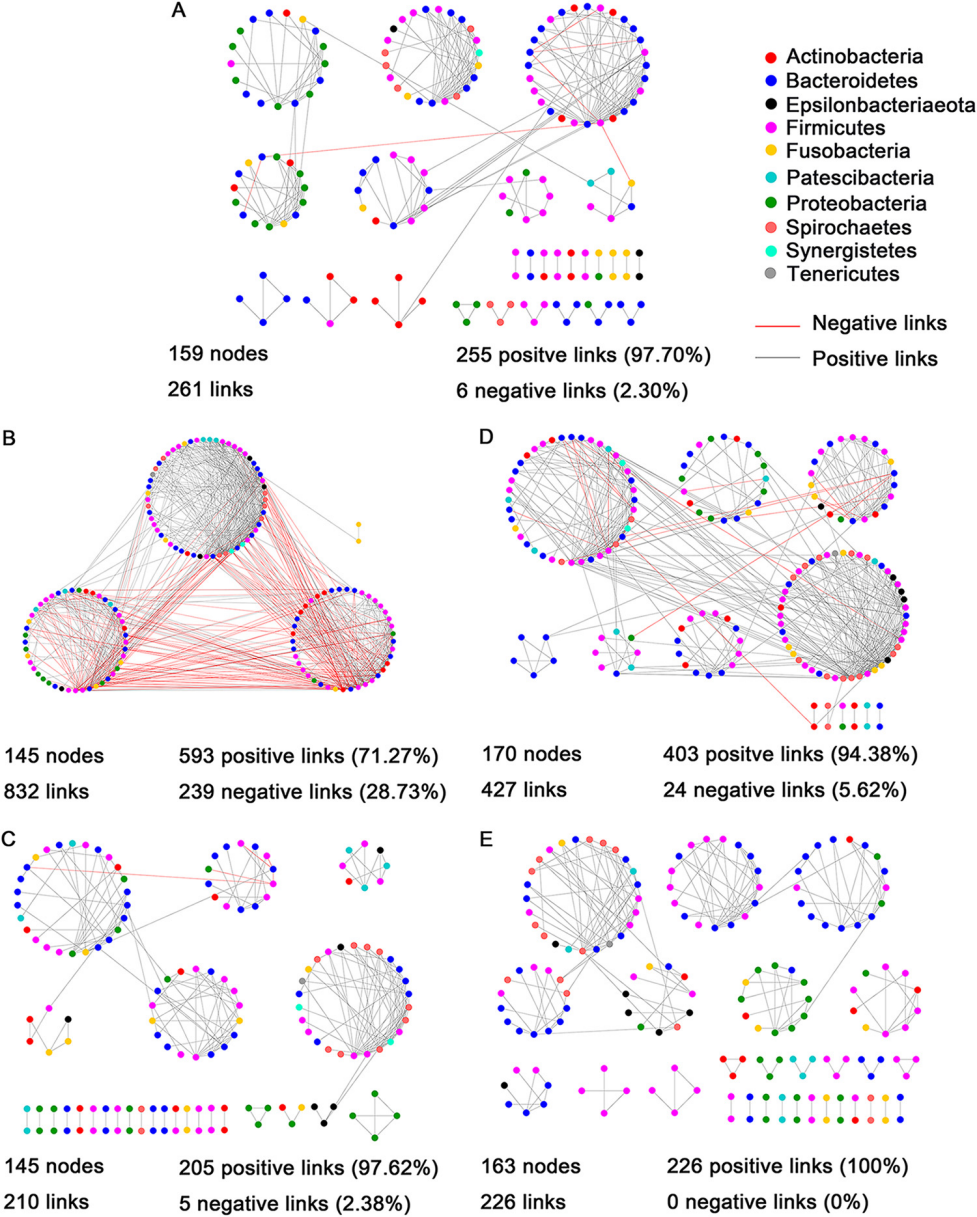
Correlation-based network analysis showing all potential interactions among the oral microbial OTUs of non-users (A), current heroin users (B), former heroin users (C), current METH users (D) and former METH users (E). The node colors indicate different phyla. The lines connecting the nodes (edges) represent positive (gray) or negative (red) cooccurrence relationships. The oral microbial networks of current drug users have a higher complexity and more negative links compared to those of non-users or former drug users.

Overall, more complex cooccurrence networks of the oral microbial communities were observed in current drug users than those in non-user and former drug users (Fig. 8, Table S6). The oral cavities of current heroin users had the most complex microbial networks with an average degree of 11.48 and a density of 0.08, followed by that of current METH users. The remaining networks had similar scales and complexities.

Furthermore, there was a larger proportion of positive correlations in the salivary networks of non-users and former drug users than in those of current drug users. The proportions of positive correlations were 97.70% (Fig. 8A), 97.62% (Fig. 8C) and 100.00% (Fig. 8E) in the networks of non-users, former heroin users and former METH users, respectively. Correspondingly, the network of current heroin users had the highest proportion of negative correlations (28.73%, Fig. 8B), which was followed by that of current METH users (5.62%, Fig. 8D).

The modularity value of each network was >0.4, which indicated that each empirical network had a modular structure (Table S6). Furthermore, the networks of non-users (0.78) and former drug user (heroin = 0.77 and METH = 0.82) had greater modularity values than those of current drug users (heroin = 0.43 and METH = 0.59), which indicated that a more obvious modular structure in oral microbial communities of non-users and former drug user. In fact, we did observe more ecological modules (e.g., each module with over six nodes) in the networks of non-users (7 modules) and former drug users (heroin: 6 modules, METH: 8 modules) than in those of current drug user (heroin: 3 modules; METH: 6 modules). These results suggested that drug addiction could decrease the modularity of the oral microbial community, especially for heroin addiction.

## DISCUSSION

In this large-scale study, we observed that the oral bacterial community compositions of drug users differed substantially from those of non-users. Regarding drug administration via nasal inhalation, smoking and direct smearing on the oral mucosa (21), drugs could have direct contact and cytotoxic effects on the oral microbial microbes. On the other hand, heroin and METH could change the oral environment, such as causing xerostomia and decreasing oral pH levels (16, 22). In addition, drug abuse may impair host immunity (23, 24). The immune system regulates relationships between the microbiota and the host, the presence of an immune system disorder can shift the microbiota by disrupting mutual or commensal relationships (25). These factors might result in changes in the bacterial community compositions and reductions in the alpha diversities in the oral cavity.

The oral microbial communities of non-users and former drug users were relatively conserved compared to current drug users. Previous studies have also shown that healthy individuals had more similar oral bacterial communities than cigarette smokers (26) and patients with dental caries (3).

The damage to the oral microbial community caused by drug abuse was not permanent. The oral microbiota of drug users could partially recover. It is well established that the oral cavity can provide highly heterogeneous ecological niches for microbes (27). This property could provide tolerance and resilience to many adverse conditions (28). The study of Wu et al. (2016) also showed that the specific microbial taxa that were altered by smoking may be restored following smoking cessation (10). However, there were still significant differences between non-users and former drug users in their oral microbial community structures. This result may imply some lingering effects of drug addiction.

Heroin and METH current users share most of the enriched and depleted OTUs compared to noncurrent ones. The descriptions in previous studies regarding the effects of heroin and METH on oral health were similar, such as xerostomia and decreases in oral pH levels (15). This may result in consistent changes in the microbial community to some extent. However, heroin had a greater effect on the oral microbial abundances and community structures. There are still differences in the oral characteristics between those two drug users. For example, METH produces a very characteristic “meth mouth,” and heroin produces dysgeusia and alterations in chewing (29). Heroin is an opiate drug, and METH is a stimulant (15). The direct toxicities of the two drugs to the oral-specific microbial taxa may be different. These influencing mechanisms might result in distinct microbial community structures.

The ages of drug user had no significant effects on the oral microbial communities before detoxication but had a significant effect after detoxication. These findings indicated that the period of drug abuse tends to influence the resilience of the oral microbial community.

A previous study observed reductions in Proteobacteria and increases in the relative abundances of Actinobacteria and Firmicutes in current cigarettes and opium smokers compared with nonsmokers (10, 30). Cigarette and cannabis smoking could decrease oral aerobes such as *Neisseria* and Haemophilus (31). Similar results were also observed in current heroin users compared to non-users and former heroin users. Heroin administration causes statistically significant reductions in the oxygen saturation rate (32). METH neurotoxicity may also be linked to changes in O_2_ levels (33). This may imply that the poor oral health status caused by different habits could exhibit similar changes in the abundances of some specific taxa.

The microbiota of population with more teeth with dental caries showed significantly lower relative abundances of *N. subflava*, *H. parainfluenzae*, *P. pasteri*, and F. periodonticum (34, 35). These decreases were also observed in the comparisons of current drug users versus non-users or former drug users. Additionally, caries-associated microbial taxa such as *P. histicola* (36) and *V. dispar* (37) enriched in oral cavities of current drug users. These oral microbial characteristics of current drug users might increase risk of dental caries. However, the decreases of *S. australis*, S. mutans, *and* S. sanguinis in current drug users were contrary to our perception. Streptococcus, especially for S. mutans, was reported to be associated with biofilm formation, which would result in greater epithelial adherence by the pathogens associated with dental caries (38, 39). This suggested that the pathogenesis of dental caries among drug users might be distinguished from non-users. In our study, most of non-users have dental caries. Further study needs to include more participants with good oral health to test the hypothesis. The study of Lee et al. (2021) based on a rodent model of prolonged drug injection and S. mutans oral infection suggested that the combination of METH and sucrose stimulates S. mutans tooth adhesion, growth, and biofilm formation (40). S. mutans tends to form biofilms in hard surfaces (teeth) rather than in tissue. Different oral sites and administration routes might be reasons to explain the opposite results. It is possible that the decreases are due to specific drug tolerance. Our study is only an observational investigation, those changed taxa whether are directly affected by drug need further exploration.

The oral microbiota has powerful classification potential for distinguishing current drug users from non-users and former drug users. The recent study of Kosciolek et al. (2021) also showed that an altered oral microbiota of individuals with substance use disorder is sufficiently distinct to accurately classify this group (14). Large numbers of crime cases are committed under the influence of drug abuse (41). The saliva strains found in crime scenes are important forensic materials (42). Our findings might provide information on the drug abuse status of individuals who left saliva in crime scene.

The ecological interactions among host-associated microbial inhabitants could influence host health and disease and would correspondingly change with the host health and disease status (43). More complex relationships were observed in the oral microbial networks of current drug users, especially the lower alpha-diversities, which suggested that drug abuse increased the interactions among microbial communities. In general, enriched resources decrease the frequency of microbial-microbial interactions and allow more microbes to maintain free-living patterns (44). The primary sources of nutrition for salivary microbial growth are not obtained from the food ingested by the host but consist of glycoproteins that are obtained from the gingival crevicular fluid and saliva (45). Therefore, the nutritional status of the host could influence the oral microbial community. The higher sugar consumption of drug users could not increase salivary nutrition since the sugar in food is quickly removed through the actions of swallowing and salivary flow (45). Drug abuse could interfere with human nutrient absorption (46). The poor nutritional status of drug users increases the interactions of the salivary microbial community. On the other hand, anaerobic metabolism with lower energy yields requires the concerted activities of various species to perform chemical transformations (47). Therefore, the oral microbial communities in the oral anaerobic environments of drug users might exhibit higher densities of interactions.

In ecological cooccurrence networks, positive correlations generally indicate synergistic (such as cooperative or syntrophic) relationships, while negative correlations indicate antagonistic (such as competitive or predation) relationships (48). The negative correlations increased by an even greater proportion in current drug users compared to non-users and former drug users. Greater nutrient limitations could increase competition (49). Violle et al. (2010) demonstrated that external disturbances could promote competitive interactions in microbial communities (50).

In cooccurrence networks, modularity contains some highly connected microbes that cluster into a group (51). In general, the nodes in various modules perform different functions (52). In our study, all networks had modular structures (modularity > 0.4) (53). The networks of current drug users had the lowest modularity values and the fewest highly connected modules, which suggest that drug abuse destroyed the healthy oral microbial ecological functions and that detoxication might contribute to the functional recovery of the oral microbial community.

In summary, we investigated the effects of drug addiction and detoxification on the oral microbial community. We observed that drug addiction may decrease the oral microbial alpha diversity and damage the balance of the oral microbial ecology. Detoxication may have positive effects on the recovery of oral microbial community structures to some extent. The strengths of this study include a sufficiently large sample size and the control of potential confounders.

We collected as many samples of current drug users as possible. In our study, participants could withdraw from the study at any time if they wish. So, we did not know if we can get samples of all these participants after detoxification (3 months). This resulted in imbalanced sampling sizes for different groups. However, we performed pairwise tests to deal with potentially bias caused by imbalanced grouping and obtained similar results with those of all samples. Additionally, synthetic train data were generated to deal with overfitting problem of imbalanced binary classification in random forest model.

In addition, the current study still has some limitations. First of all, the small size of female sample was a limitation of our study, especially for former drug users. Second, all participants are cigarette smokers, most of them have oral diseases. However, we failed to get the more detailed data such as prevalence rate of decayed and filled teeth and the mean decayed and filled teeth score. Future studies should cover a larger of samples, including more females and oral health populations. And more detailed oral health status was essential to explore the relationships between drug addicting, oral health status and oral microbiota. Finally, the current study is only an observational investigation and is not sufficient to determine the functional and gene contents of bacteria that are altered by drug addiction and detoxification. Further studies should explore the impacts of drug addiction and detoxification on the oral microbiome by using metagenomic data and whether addiction-related oral microbiomes mediate the health of individuals with drug addiction.

## MATERIALS AND METHODS

### Study population.

The study has received ethics approval from the Shanxi Medical University of Medicine Institutional Review Board (no. 2021GLL049), in accordance with the guidelines of the World Medical Association and the Helsinki Declaration. The participants were obtained from Chongqing. All participants gave written informed consent to participation in the study. Oral wash samples were collected from 186 heroin users and 109 METH users before detoxification. We also collected oral wash samples from drug users after detoxification (71 heroin users and 79 METH users). Among them, there were 55 heroin users and 61 METH users who had samples, including both before and after detoxification (Fig. 1). The inclusion conditions of users all reached the criteria of Diagnostic and Statistical Manual of Mental Disorders (DSM-V) (54). The heroin formulation in our study is powder. The main routes of heroin administration were smoking and injection. All injecting drug users were also smoking ones. And the route of METH in our study were smoking. In addition, 50 non-users were recruited from the local region for use as controls. We recorded the age, sex, height, weight, BMI, cigarette smoking status, and oral health status of the participants (Table 1). All participants in our study are cigarette smokers. Oral health status, including statuses of dental caries and periodontal disease, were directly examined by the dentists at the sampling site. The status of dental caries was categorized as having caries or not. And periodontal status was classified as suspected of having periodontitis or not. Specifically, a participant was categorized as suspected of having periodontitis who had a depth of gingivitis pocket ≥4 mm or gum bleeding.

**TABLE 1:** Parameters, including age, sex, height, weight, BMI, smoking status, oral health status, addicting age and frequency of different groups^*a*^

Characteristics	Non-users	Current heroin users	Former heroin users	Current METH users	Former METH users
	(*n* = 50)	(*n* = 186)	(*n* = 71)	(*n* = 109)	(*n* = 79)
Sex = Male (%)	48 (96%)	175 (94.09%)	71 (100%)	104 (95.41%)	79 (100%)
Age (yrs, mean ± SD)	39.76 ± 10.21	46.25 ± 8.34***	45.63 ± 7.89**	37.18 ± 10.63	35.71 ± 9.25*
Ht (cm, mean ± SD)	169.70 ± 5.78	167.36 ± 6.76	168.07 ± 5.48	169.26 ± 6.10	169.89 ± 4.70
Wt (kg, mean ± SD)	64.41 ± 12.85	59.06 ± 10.23*	59.51 ± 8.09	65.77 ± 11.54	65.47 ± 9.87
BMI (kg/cm^2^, mean ± SD)	22.25 ± 3.59	21.03 ± 3.04	21.05 ± 2.53	22.79 ± 4.42	22.71 ± 3.50
Age of Cigarette smoking (yrs, mean ± SD)	18.67 ± 8.45	24.79 ± 10.29*	25.39 ± 8.62**	18.11 ± 10.76	17.68 ± 9.62*
Frequency of cigarette smoking (times/day, mean ± SD)	20.51 ± 9.42	20.87 ± 8.62	20.97 ± 8.43	16.65 ± 8.61*	17.36 ± 8.86*
Periodontal disease (%)	90%	98.92%	97.18%	99.08%	98.73%
Dental caries (%)	96%	100%	100%	100%	100%
Age of drug addiction (yrs, mean ± SD)	0.00 ± 0.00	16.05 ± 7.11***	16.17 ± 6.62***	5.65 ± 5.64***	6.35 ± 4.89***
Frequency of drug addicting (times/mo., mean ± SD)	0.00 ± 0.00	45.84 ± 32.12 ***	41.39 ± 30.89 ***	5.43 ± 5.21***	6.14 ± 5.81***

a *, *P* < 0.05; **, *P* < 0.01; and ***, *P* < 0.001 compared with non-users. BMI, body mass index. Frequency of cigarette smoking and frequency of drug addiction are the data of recent six mouths.

The 379 participants were asked to collect saliva samples using a sterile specimen tube by the simple drooling method. We refrained the participants from eating, drinking and brushing their teeth 1 h prior to collection. We excluded those individuals who reported antibiotic or prescribed probiotic use in the previous 3 months. The collected samples were stored at −80°C until use.

### Bacterial community analysis.

Total genomic DNA was extracted from salivary samples using the DNeasy PowerSoil kit by following the manufacturer’s instructions. Sequencing libraries were prepared by following the Illumina 16S Metagenomic Sequencing Library Preparation workflow. The V3-V4 regions of the 16S rRNA gene were amplified with the bacterial universal primer combination (341F-5′-CCTACGGGNGGCWGCAG-3′ and 806R-5′- GGACTACHVGGGTWTCTAAT-3′), which contained a barcode sequence for Illumina MiSeq sequencing from each sample (55). The PCR products were pooled and cleaned using the Qiagen Gel Extraction kit (Qiagen, Hilden, Germany) and were then sequenced with the Illumina MiSeq FGX platform. Negative controls consisting of sterile water were included during DNA extraction and sequencing.

The raw 16S rRNA gene amplicon sequences were merged, quality filtered and clustered by using a combination of QIIME (56), VSEARCH (57) and USEARCH (58). Chimeric sequences were removed based on the UCHIME algorithm (59). The sequences were clustered into operational taxonomic units (OTUs) with 97% similarity. Following these steps, the OTU sequences were obtained. The taxonomic assignment of the representative OTU sequences was annotated by matching the Human Oral Microbiome Database (eHOMD 16S rRNA RefSeq Version 15.22) (60).

### Statistical analysis.

The data analyses were conducted using the R software platform. The standard sampling sizes were estimated based on Cohen D values of alpha diversity (phylogenetic diversity, PD) as the method of Casals-Pascual et al. (2020) (61). The alpha diversity of each sample was expressed as the number of observed OTUs, Shannon index and phylogenetic diversity (PD) by using a rarified OTU table. The beta diversities were determined by the Bray–Curtis and weighted UniFrac distances. The significant differences in taxa-relative abundances, alpha diversities and beta diversities among various groups were tested by using the Wilcoxon test with *P* values adjusted according to the Benjamini-Hochberg method. Principal coordinate analysis was performed using the “pcoa” function in the “ape” package (62), and the first two principal coordinates were presented in a visualization. A permutational multivariate analyses of variance (PERMANOVA) based on the Bray–Curtis and weighted UniFrac distances was used to determine the differences in bacterial compositions across the drug addiction status categories and were adjusted for age, sex, height, weight, BMI, cigarette smoking status and oral health status. The variance estimations for the OTUs among different drug addiction statuses were determined by fitting a negative binomial generalized linear model using the “edgeR” R package (63).

We used the random forest classifier to select microbial biomarkers to distinguish the never, former and current addiction statuses at the species level using the “randomForest” R package (64). The train and test sets were build using stratified random sampling method as the proportion of 2:1 by the “sampling” R package (65). The ROSE package was employed to generate synthetic train data for dealing with overfitting problem of imbalanced binary classification according to a bootstrapping approach (66). The species were ranked in order of their feature importance in the random forest model by using 100 iterations. Five repeats of 10-fold cross-validation were performed to select the optimal classifiers. Then, random forest models were established based on the selected optimal classifiers. ROC curves were obtained for evaluations of the random forest models based on the selected optimal markers (67).

Network construction was accomplished with the Cytoscape plugin CoNet (68) by using an ensemble-based approach that combined two measures of correlation (e.g., Pearson and Spearman) and dissimilarity (e.g., Bray–Curtis and Kullback–Leibler). The bacterial OTUs that occurred in <1/3 of the salivary samples were removed. Then, we requested the 1000 top- and bottom-ranking links for each statistical measure. The statistical significances were calculated by obtaining the method- and edge-specific permutation and bootstrap score distributions using 1000 iterations for each distribution. The *P* values were merged using Brown’s method and were corrected for multiple tests by using the Benjamini–Hochberg procedure; the obtained networks were filtered to preserve only those links with adjusted merged *P* values < 0.001. Further statistical analyses of these networks were conducted with the implemented tool, NETWORKANALYZER (69), and the igraph R package (70).

### Data availability.

Sequencing data are deposited at the Genome Sequence Archive in the BIG Data Center, Chinese Academy of Sciences, under accession number CRA005827.
