# Supplementary material for: The Effects of Drug Addiction and Detoxification on the Human Oral Microbiota
Source: Microbiol Spectr. 2023 Feb 1;11(2):e03961-22. doi: 10.1128/spectrum.03961-22 (PMC10100366; doi:10.1128/spectrum.03961-22)
Supplement: Supplemental file 1 — Supplemental material. Download spectrum.03961-22-s0001.pdf, PDF file, 3.2 MB [file spectrum.03961-22-s0001.pdf]

Table S1 PERMANOVA testing the effects of drug addicting categories on oral bacterial Bray-Curtis distance after adjusting for age, sex, height, weight, BMI, smoking status and oral health status.

	Non-users vs. Current heroin users		Non-users vs. Former heroin users		Non-users vs. Current METH users		Non-users vs. Former METH users		Current heroin users vs. Former heroin users		Current METH users vs. Former METH users		Current heroin users vs. Current METH users		Former heroin users vs. Former METH users	
	R^2	P	R^2	P	R^2	P	R^2	P	R^2	P	R^2	P	R^2	P	R^2	P
Sex	**0.023**	**0.001**	0.011	0.127	**0.020**	**0.001**	0.012	0.101	**0.029**	**0.001**	**0.017**	**0.001**	**0.028**	**0.001**	NA	NA
Age	**0.020**	**0.001**	**0.019**	**0.001**	**0.023**	**0.001**	**0.017**	**0.006**	**0.011**	**0.004**	**0.017**	**0.001**	**0.031**	**0.001**	**0.020**	**0.001**
Age of Cigarette smoking	0.004	0.412	0.008	0.430	0.006	0.316	0.011	0.083	0.003	0.546	0.006	0.209	0.003	0.495	0.009	0.113
Frequency of Cigarette smoking	0.004	0.592	0.009	0.358	**0.012**	**0.005**	0.008	0.386	0.004	0.249	0.005	0.281	0.003	0.457	0.005	0.964
Periodontal diseases	0.006	0.069	0.006	0.937	**0.012**	**0.020**	0.007	0.510	0.004	0.405	0.005	0.449	0.002	0.782	0.006	0.707
Dental caries	0.006	0.084	0.011	0.143	0.009	0.094	0.010	0.190	NA	NA	NA	NA	NA	NA	NA	NA
Height	0.005	0.295	0.010	0.265	0.008	0.116	0.009	0.244	0.004	0.346	0.008	0.102	0.003	0.356	0.009	0.134
Weight	0.003	0.751	0.009	0.291	0.005	0.531	0.009	0.320	0.002	0.947	0.005	0.389	0.003	0.584	0.005	0.832
BMI	**0.007**	**0.033**	0.011	0.141	0.008	0.132	**0.018**	**0.002**	**0.007**	**0.023**	**0.010**	**0.010**	0.004	0.263	0.010	0.056
Drug addicting categories	**0.066**	**0.001**	**0.033**	**0.001**	**0.118**	**0.001**	**0.031**	**0.001**	**0.087**	**0.001**	**0.129**	**0.001**	**0.027**	**0.001**	0.009	0.104

Note: Bold letters indicate significant differences ($P < 0.05$). Abbreviations: BMI, body mass index. Frequency of cigarette smoking is the data of recent six mouths.

Table S2 PERMANOVA testing the effects of drug addicting categories on oral bacterial Weighted Unifrac distance after adjusting for age, sex, height, weight, BMI, smoking status and oral health status.

	Non-users vs. Current heroin users		Non-users vs. Former heroin users		Non-users vs. Current METH users		Non-users vs. Former METH users		Current heroin users vs. Former heroin users		Current METH users vs. Former METH users		Current heroin users vs. Current METH users		Former heroin users vs. Former METH users	
	R^2	P	R^2	P	R^2	P	R^2	P	R^2	P	R^2	P	R^2	P	R^2	P
Sex	**0.033**	**0.001**	**0.022**	**0.016**	**0.033**	**0.001**	0.019	0.056	**0.039**	**0.001**	**0.023**	**0.003**	**0.040**	**0.001**	NA	NA
Age	**0.019**	**0.002**	**0.017**	**0.048**	**0.029**	**0.001**	0.014	0.079	**0.012**	**0.009**	**0.021**	**0.001**	**0.042**	**0.001**	0.012	0.092
Age of Cigarette smoking	0.004	0.421	0.006	0.695	0.006	0.310	0.012	0.147	0.003	0.653	0.006	0.331	0.003	0.343	0.012	0.079
Frequency of Cigarette smoking	0.005	0.263	0.011	0.183	0.011	0.056	0.010	0.208	0.004	0.377	0.003	0.797	0.003	0.413	0.004	0.895
Periodontal diseases	0.004	0.391	0.004	0.949	0.009	0.144	0.006	0.564	0.004	0.379	0.003	0.657	0.002	0.609	0.005	0.531
Dental caries	0.006	0.170	0.010	0.269	0.010	0.068	0.009	0.255	NA	NA	NA	NA	NA	NA	NA	NA
Height	0.007	0.129	0.008	0.419	0.005	0.499	**0.017**	**0.039**	0.006	0.102	0.005	0.326	0.002	0.740	0.015	0.026
Weight	0.001	0.964	0.006	0.633	0.003	0.908	0.010	0.187	0.002	0.765	0.002	0.983	0.002	0.769	0.004	0.852
BMI	0.009	0.034	0.011	0.212	0.008	0.183	**0.019**	**0.032**	0.008	0.055	**0.012**	**0.015**	0.006	0.145	0.013	0.062
Drug addicting categories	**0.059**	**0.001**	**0.043**	**0.001**	**0.127**	**0.001**	**0.040**	**0.001**	**0.070**	**0.001**	**0.090**	**0.001**	**0.032**	**0.001**	0.005	0.611

Note: Bold letters indicate significant differences ($P < 0.05$). Abbreviations: BMI, body mass index. Frequency of cigarette smoking is the data of recent six mouths.

Table S3 PERMANOVA testing the effects of drug addicting categories on oral bacterial Bray-Curtis and Weighted Unifrac distances using pair-wise data after adjusting for age, sex, height, weight, BMI, smoking status and oral health status.

	Bray-Curtis distance				Weighted Unifrac distance			
	Current heroin users vs. Former heroin users		Current METH users vs. Former METH users		Current heroin users vs. Former heroin users		Current METH users vs. Former METH users	
	R^2	P	R^2	P	R^2	P	R^2	P
Age	**0.016**	**0.019**	**0.019**	**0.005**	**0.017**	**0.033**	**0.018**	**0.016**
Age of Cigarette smoking	0.012	0.057	**0.017**	**0.013**	0.013	0.092	**0.025**	**0.004**
Frequency of Cigarette smoking	0.011	0.109	0.007	0.332	0.009	0.312	0.005	0.660
Periodontal diseases	0.008	0.454	0.007	0.341	0.005	0.796	0.011	0.537
Height	**0.014**	**0.048**	0.008	0.250	0.016	0.036	0.006	0.007
Weight	0.011	0.139	0.008	0.198	0.009	0.375	0.006	0.491
BMI	0.008	0.380	0.011	0.059	0.005	0.838	0.015	0.044
Drug addicting categories	**0.160**	**0.001**	**0178**	**0.001**	0.129	0.001	**0.145**	**0.001**

Note: Bold letters indicate significant differences ($P < 0.05$). Abbreviations: BMI, body mass index. Frequency of cigarette smoking is the data of recent six mouths.

Table S4 PERMANOVA testing the effects of age of drug addiction on oral bacterial Bray-Curtis and Weighted Unifrac distances after adjusting for age, sex, height, weight, BMI, smoking status and oral health status.

	Bray-Curtis distance								Weighted Unifrac distance							
	Current heroin		Former heroin		Current METH		Former METH		Current heroin		Former heroin		Current METH		Former METH	
	R^2	P	R^2	P	R^2	P	R^2	P	R^2	P	R^2	P	R^2	P	R^2	P
Sex	**0.023**	**0.001**	NA	NA	**0.035**	**0.001**	NA	NA	**0.031**	**0.001**	NA	NA	**0.051**	**0.001**	NA	NA
Age	**0.011**	**0.017**	0.022	0.052	**0.025**	**0.004**	0.019	0.052	**0.013**	**0.021**	0.025	0.089	**0.032**	**0.005**	0.012	0.431
Age of Cigarette smoking	0.005	0.578	0.014	0.514	0.009	0.485	0.019	0.072	0.004	0.547	0.013	0.554	0.009	0.496	0.024	0.081
Frequency of Cigarette smoking	0.005	0.661	0.015	0.421	0.008	0.659	0.009	0.889	0.005	0.483	0.012	0.567	0.005	0.815	0.005	0.930
Periodontal diseases	0.005	0.544	0.014	0.752	0.006	0.863	0.010	0.563	0.005	0.488	0.008	0.853	0.005	0.810	0.011	0.319
Height	0.005	0.529	0.0.013	0.643	0.014	0.128	0.017	0.114	0.008	0.204	0.008	0.860	0.013	0.225	**0.038**	**0.007**
Weight	0.003	0.987	0.012	0.714	0.009	0.406	0.009	0.883	0.002	0.960	0.009	0.785	0.006	0.805	0.005	0.943
BMI	0.010	0.043	0.011	0.876	**0.017**	**0.022**	**0.022**	**0.041**	0.012	0.043	0.011	0.662	0.017	0.080	0.026	0.075
Age of drug addiction	0.006	0.381	**0.028**	**0.004**	0.015	0.070	**0.022**	**0.032**	0.005	0.509	**0.040**	**0.009**	0.018	0.054	**0.022**	**0.096**

Note: Bold letters indicate significant differences ($P < 0.05$). Abbreviations: BMI, body mass index. Frequency of cigarette smoking is the data of recent six mouths.

Table S5 PERMANOVA testing the effects of frequency of drug addicting on oral bacterial Bray-Curtis and Weighted Unifrac distances after adjusting for age, sex, height, weight, BMI, smoking status and oral health status.

	Bray-Curtis distance								Weighted Unifrac distance							
	Current heroin		Former heroin		Current METH		Former METH		Current heroin		Former heroin		Current METH		Former METH	
	R^2	P	R^2	P	R^2	P	R^2	P	R^2	P	R^2	P	R^2	P	R^2	P
Sex	**0.023**	**0.001**	NA	NA	**0.038**	**0.001**	NA	NA	**0.029**	**0.001**	NA	NA	**0.055**	**0.002**	NA	NA
Age	**0.012**	**0.013**	0.024	0.053	**0.025**	**0.009**	0.021	0.039	**0.014**	**0.037**	0.028	0.087	**0.032**	**0.002**	0.014	0.364
Age of Cigarette smoking	0.005	0.740	0.017	0.357	0.009	0.617	0.020	0.071	0.004	0.713	0.016	0.460	0.009	0.497	0.026	0.068
Frequency of Cigarette smoking	0.005	0.740	0.014	0.713	0.008	0.635	0.009	0.908	0.005	0.493	0.014	0.568	0.006	0.831	0.006	0.881
Periodontal diseases	0.005	0.502	0.012	0.770	0.006	0.860	0.010	0.629	0.006	0.424	0.009	0.866	0.005	0.838	0.011	0.333
Height	0.006	0.447	0.015	0.534	0.015	0.127	0.019	0.117	0.009	0.151	0.009	0.852	0.014	0.232	**0.042**	**0.016**
Weight	0.003	0.954	0.015	0.562	0.011	0.303	0.009	0.923	0.003	0.848	0.009	0.876	0.009	0.507	0.004	0.994
BMI	**0.010**	**0.039**	0.013	0.769	0.014	0.110	0.022	0.053	**0.013**	**0.048**	0.013	0.642	0.013	0.211	0.026	0.085
Frequency of drug addicting	0.005	0.616	0.020	0.151	**0.018**	**0.023**	0.017	0.152	0.004	0.649	0.024	0.155	0.014	0.164	0.024	0.088

Note: Bold letters indicate significant differences ($P < 0.05$). Abbreviations: BMI, body mass index. Frequency of cigarette smoking and frequency of drug addiction are the data of recent six mouths.

Table S6 Parameters of the oral microbial co-occurrence networks of different categories of addicting status.

	No. of nodes	No. of links	Clustering coefficient	Average shortest path length	Diameter	Modularity	Degree	Density
Non-users	159	261	0.52	5.38	17	0.78	3.28	0.021
Current heroin users	145	832	0.55	2.81	7	0.43	11.48	0.080
Former heroin users	145	210	0.46	4.62	13	0.77	2.90	0.020
Current METH users	170	427	0.50	4.36	11	0.59	5.02	0.030
Former METH users	163	226	0.50	4.05	12	0.82	2.77	0.017

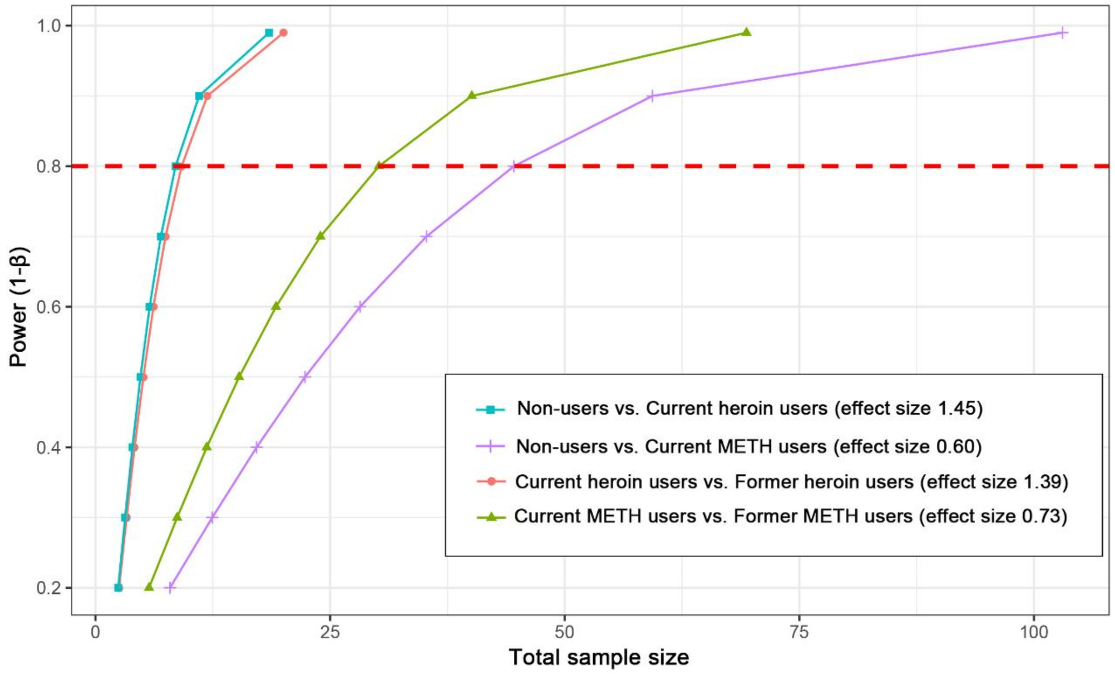

Fig. S1. Standard sample size calculation. Number of participants required to find significant differences in alpha-diversity (PD) between current drug-users and noncurrent ones, statistical power (80%) for 4 effect sizes (a difference of PD of non-users versus current heroin users, non-users versus current METH users, current heroin users versus former heroin users and current METH users versus former METH users which correspond with a Cohen D of approximately 1.45, 0.60, 1.39 and 0.73, respectively). Required sample sizes of each group are 9, 45, 10 and 31 in these comparisons, respectively. Our sample sizes exceed the standard sample size.

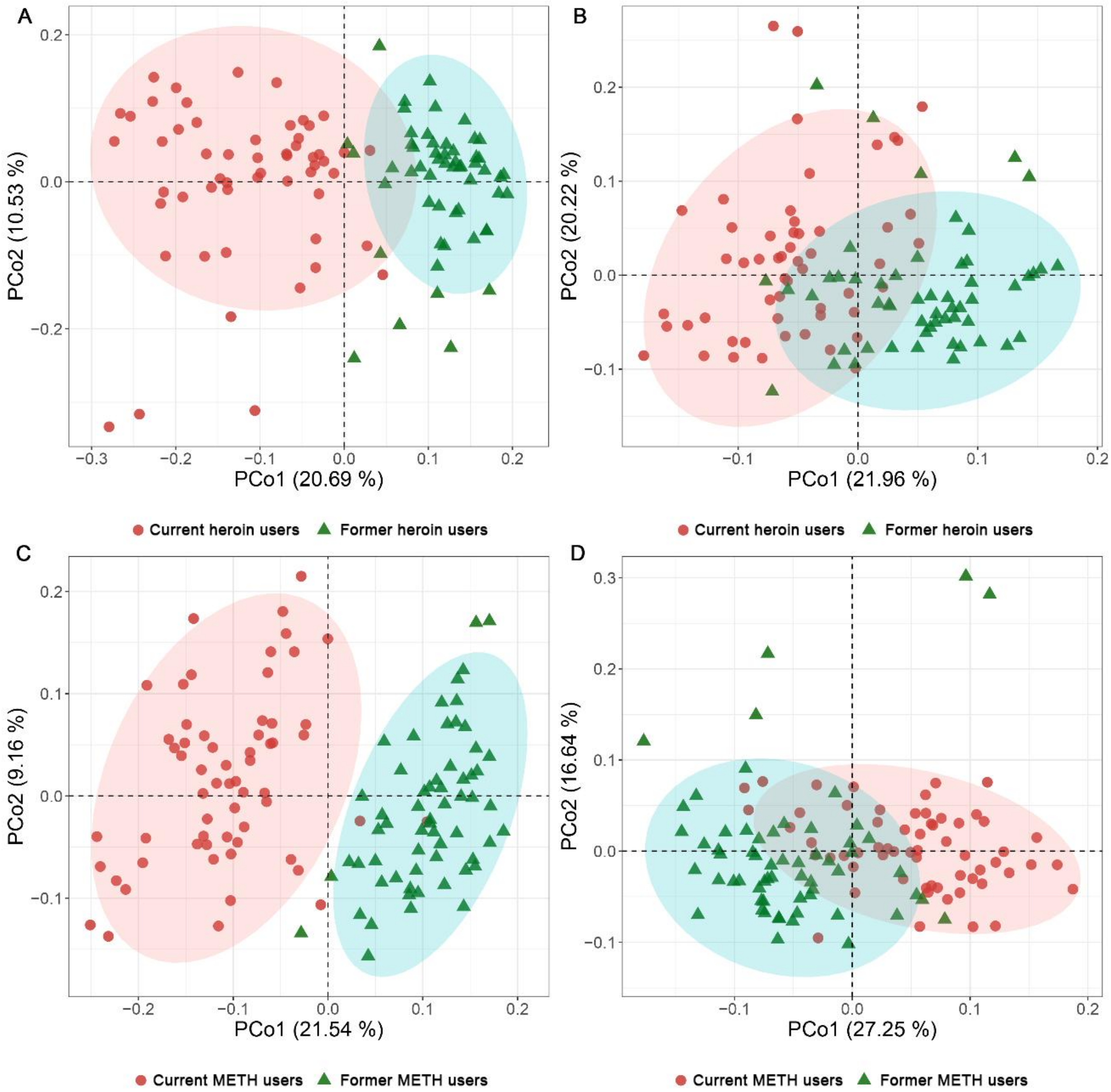

Fig. S2. Principal coordinate analysis based on the Bray–Curtis (A and C) and weighted UniFrac (B and D) distances using pair-wise data showing the contribution of drug (A and B for heroin; C and D for METH) addicting statuses (current and former) to shaping the compositions of oral microbial communities.

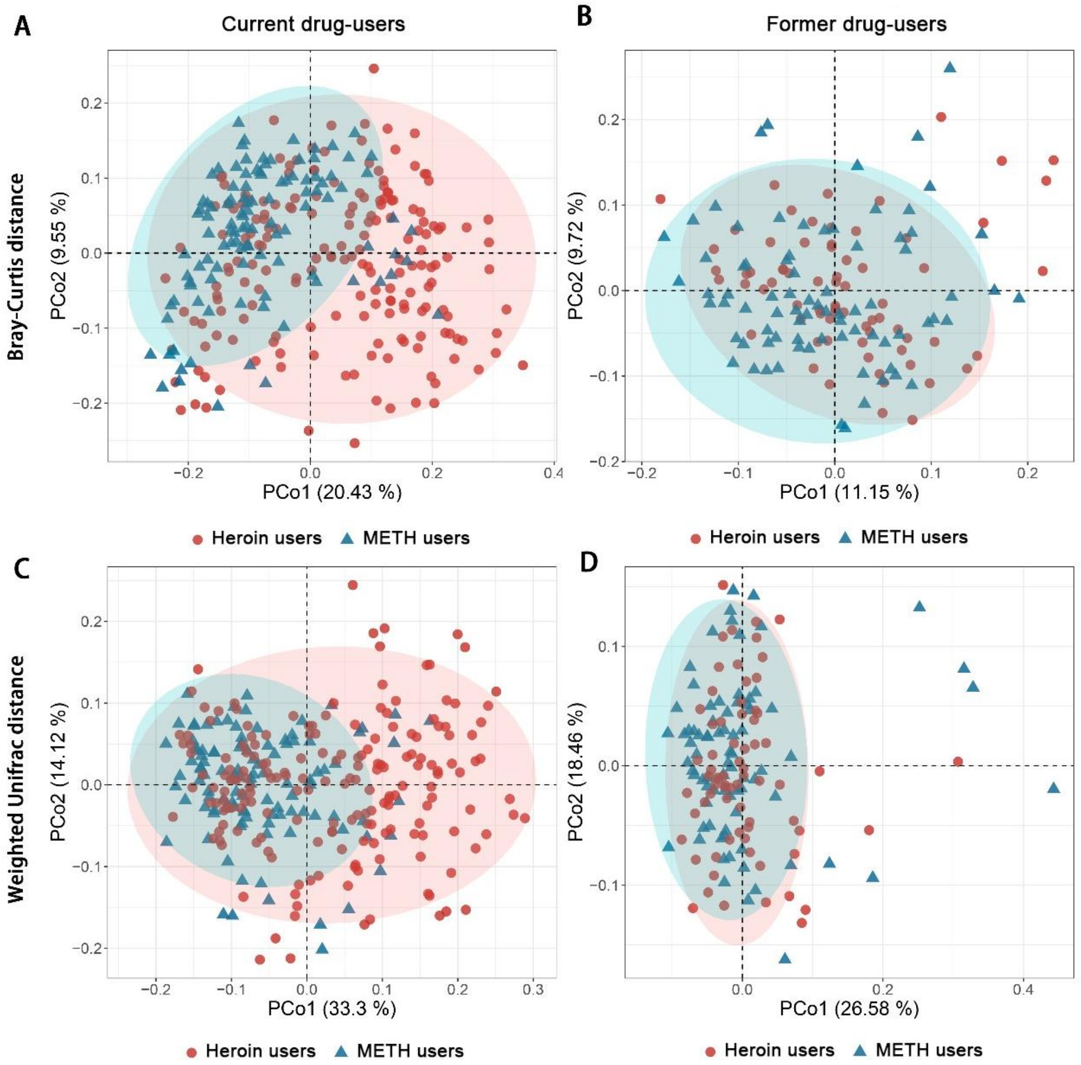

Fig. S3. Principal coordinate analysis based on Bray-Curtis (A and B) and weighted UniFrac (C and D) distances the showing contribution of drug addicting types (heroin and METH) on shaping the composition of oral microbial communities of current drug-users (A and C) and former drug-users (B and D).

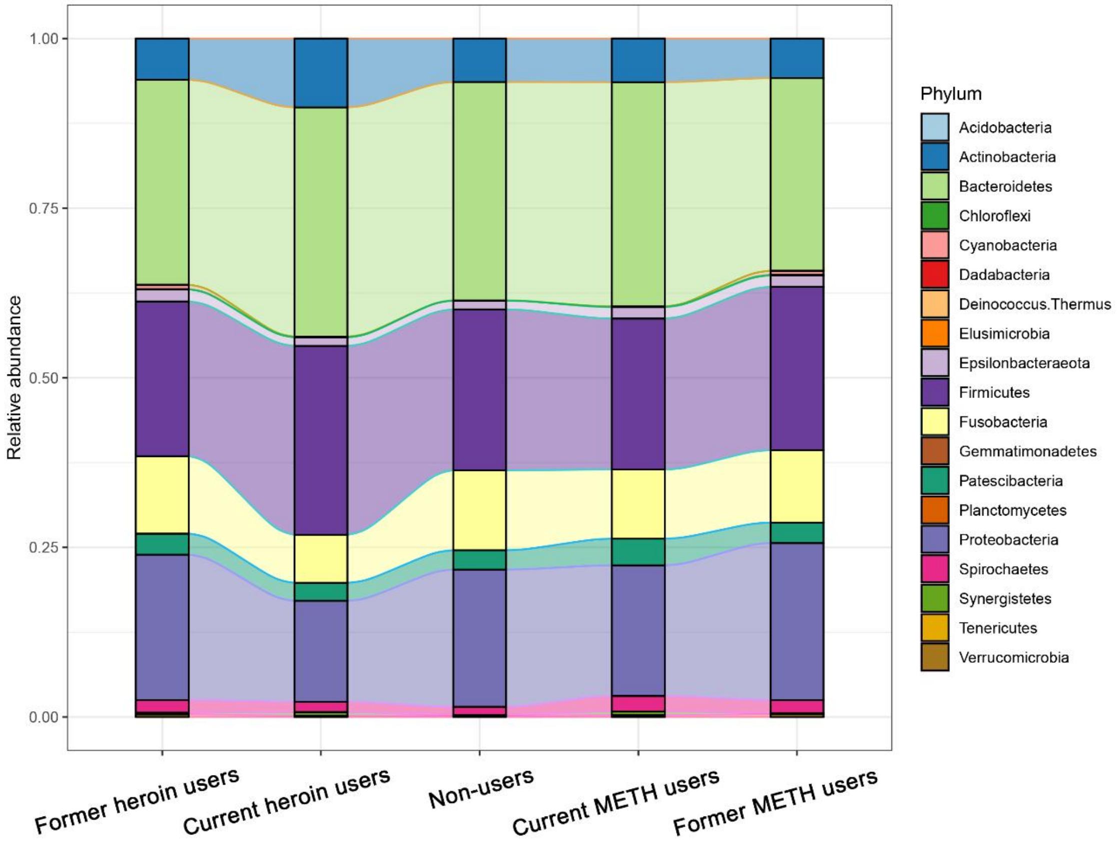

Fig. S4. Relative abundances of the phyla of the oral microbiology for various addiction categories.

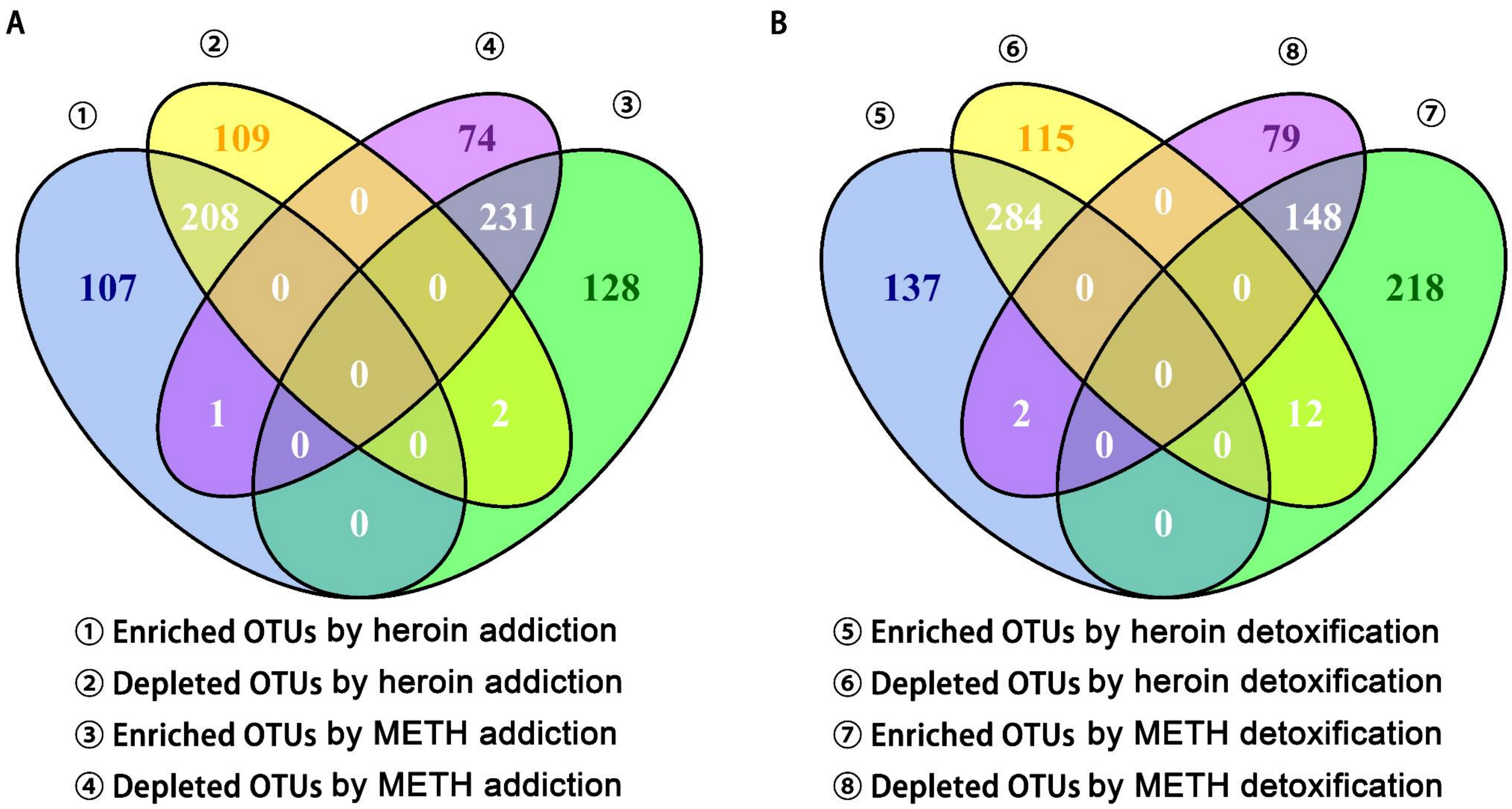

Fig. S5. Venn diagrams depicting the number of OTUs enriched and depleted caused by heroin and METH addiction (A) and detoxification (B).

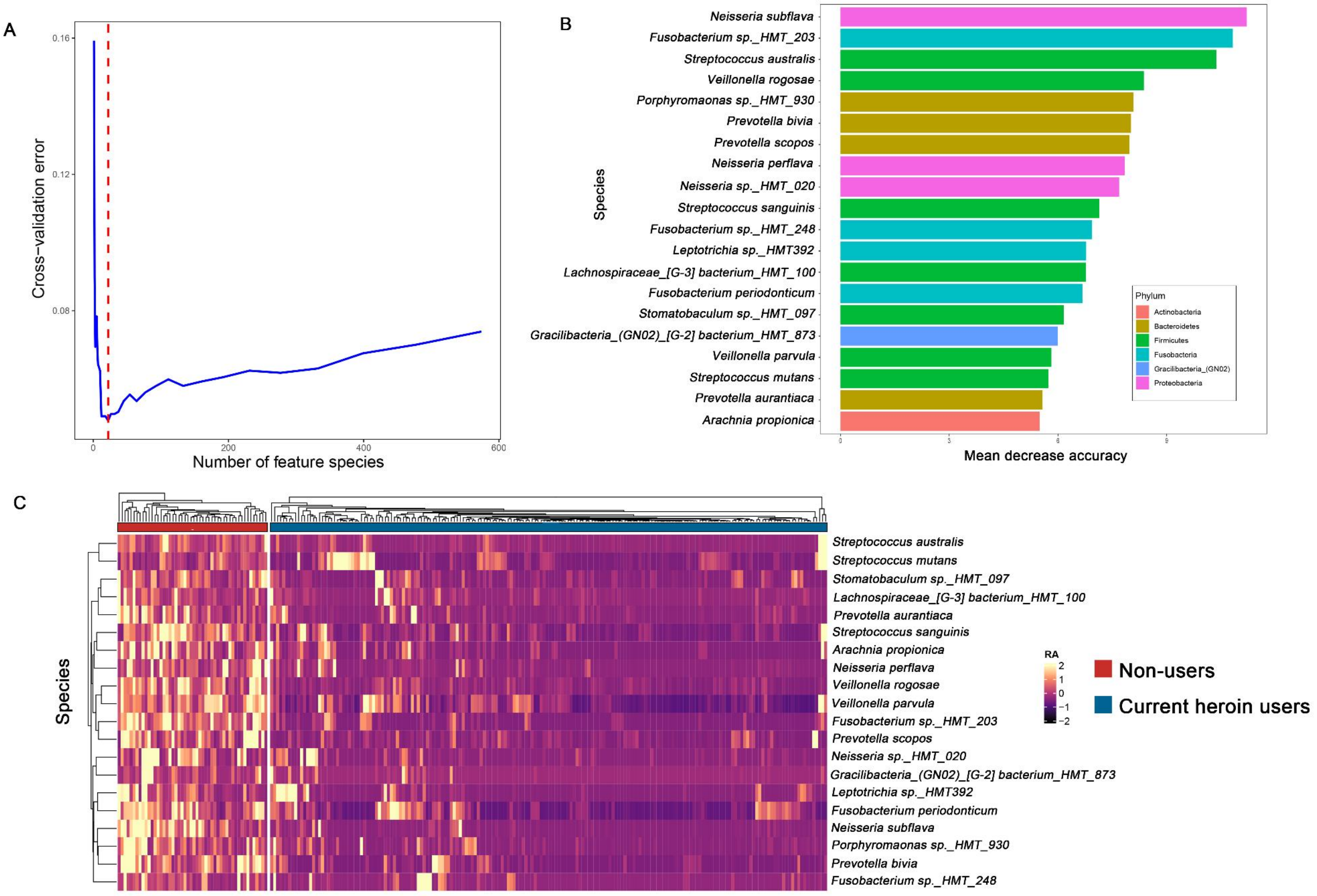

Fig. S6. Oral bacterial taxonomic biomarkers to distinguish current heroin users and non-users at the level of species. The top 22 biomarker bacterial species were identified by applying Random Forest classifier based on minimum value of 10-fold cross-validation error (A). And the top 20 biomarker taxa are ranked in descending order of importance to the accuracy of the model (B). The heatmap (the values were Z-score transformed) shows that the relative abundances of the top 20 biomarker taxa in the optimal markers when comparing current heroin users with non-users (C).

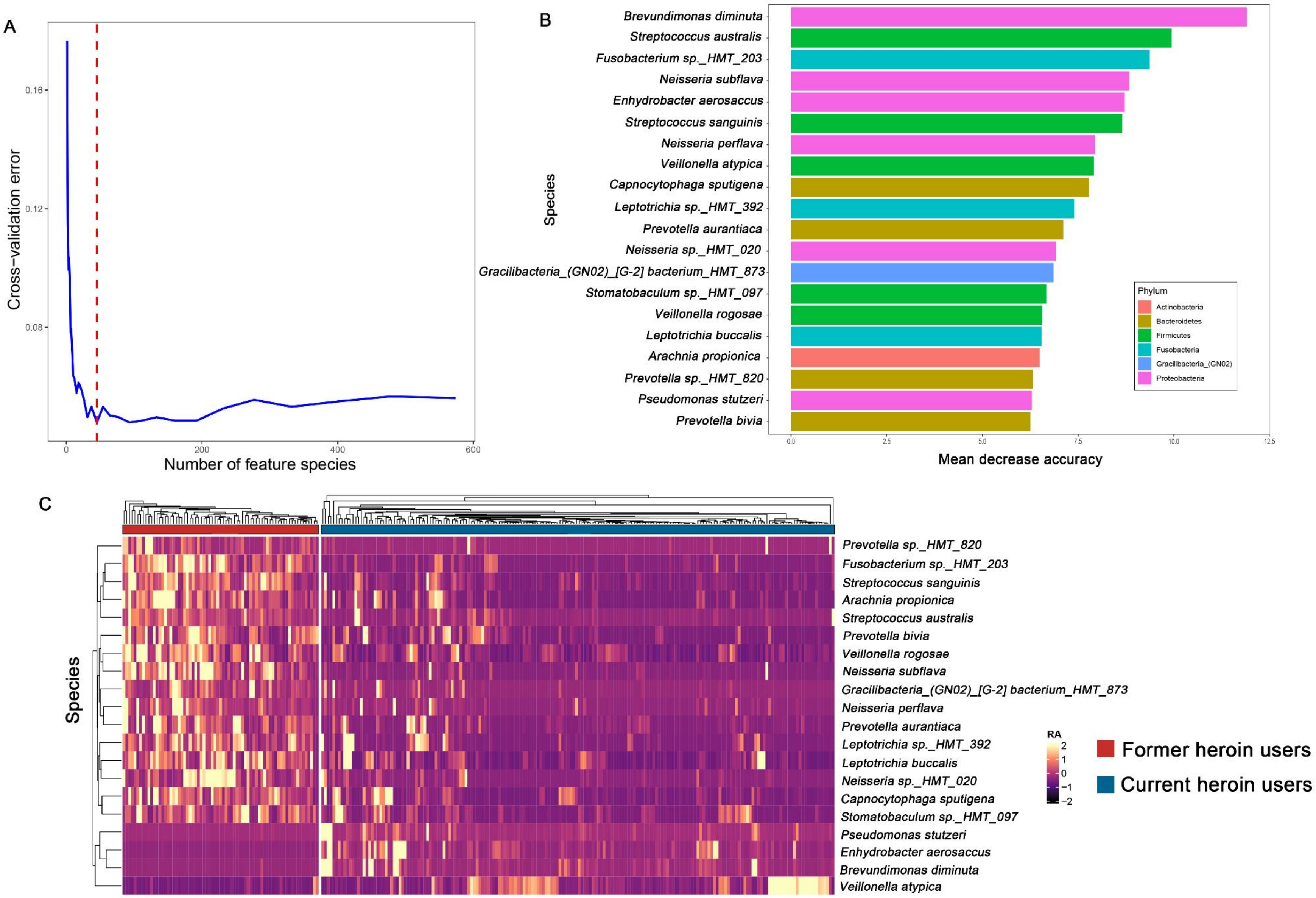

Fig. S7. Oral bacterial taxonomic biomarkers to distinguish current heroin users and former heroin users at the level of species. The top 45 biomarker bacterial species were identified by applying Random Forest classifier based on minimum value of 10-fold cross-validation error (A). And the top 20 biomarker taxa are ranked in descending order of importance to the accuracy of the model (B). The heatmap (the values were Z-score transformed) shows that the relative abundances of the top 20 biomarker taxa in the optimal markers when comparing current heroin users with former heroin users (C).

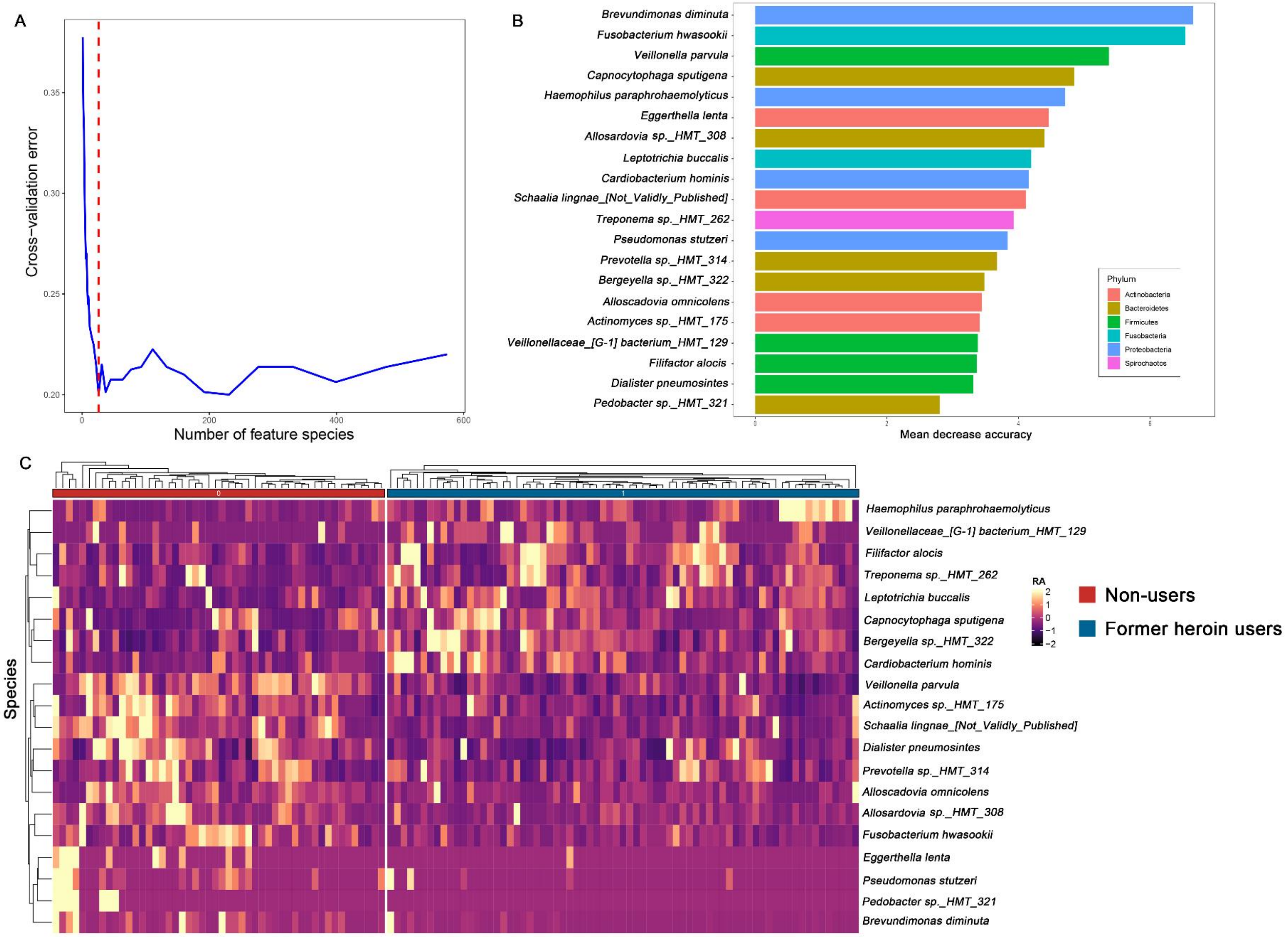

Fig. S8. Oral bacterial taxonomic biomarkers to distinguish former heroin users and non-users at the level of species. The top 26 biomarker bacterial species were identified by applying Random Forest classifier based on minimum value of 10-fold cross-validation error (A). And the top 20 biomarker taxa are ranked in descending order of importance to the accuracy of the model (B). The heatmap (the values were Z-score transformed) shows that the relative abundances of the top 20 biomarker taxa in the optimal markers when comparing former heroin users with non-users (C).

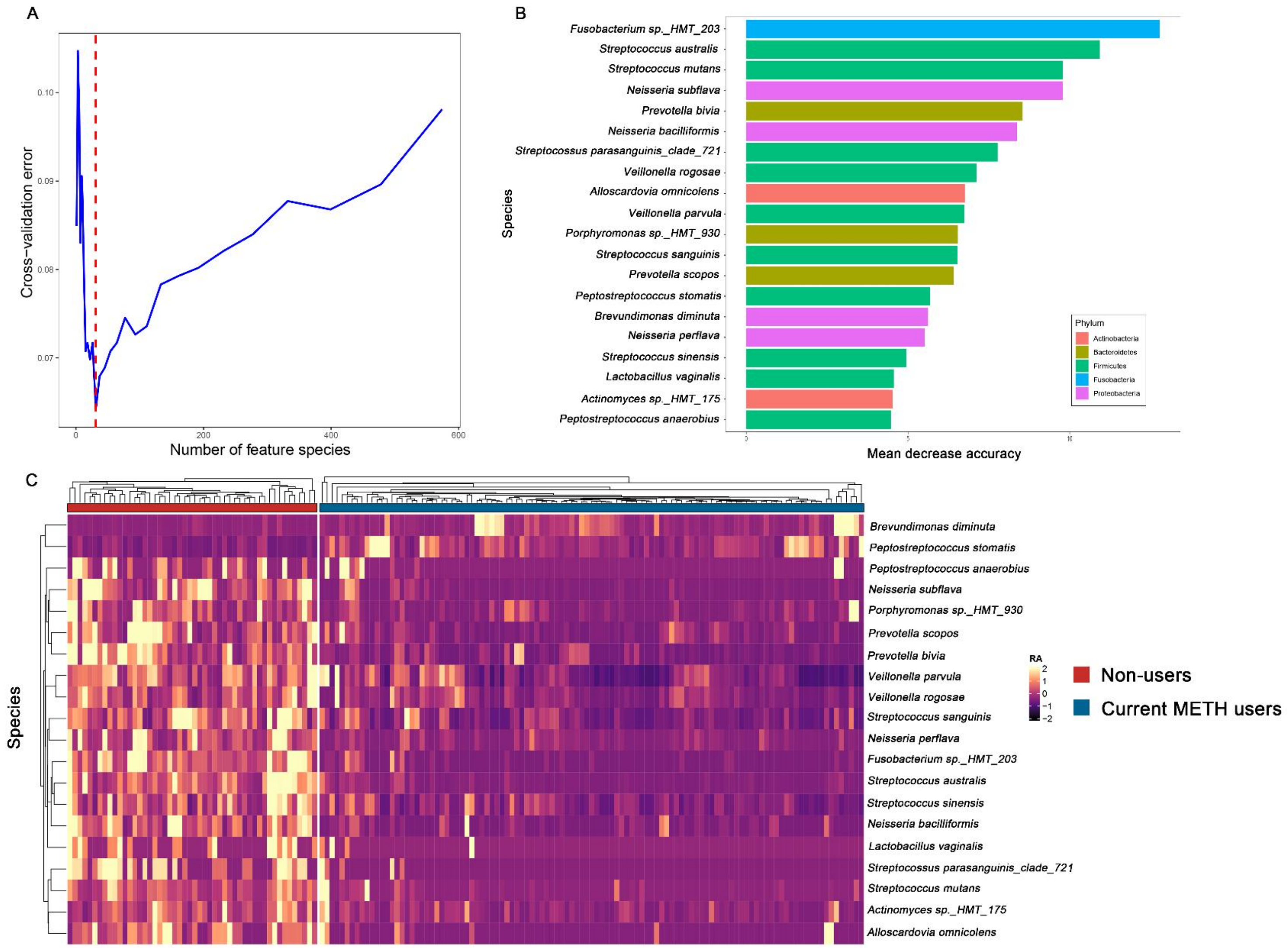

Fig. S9. Oral bacterial taxonomic biomarkers to distinguish current METH users and non-users at the level of species. The top 31 biomarker bacterial species were identified by applying Random Forest classifier based on minimum value of 10-fold cross-validation error (A). And the top 20 biomarker taxa are ranked in descending order of importance to the accuracy of the model (B). The heatmap (the values were Z-score transformed) shows that the relative abundances of the top 20 biomarker taxa in the optimal markers when comparing current METH users with non-users (C).

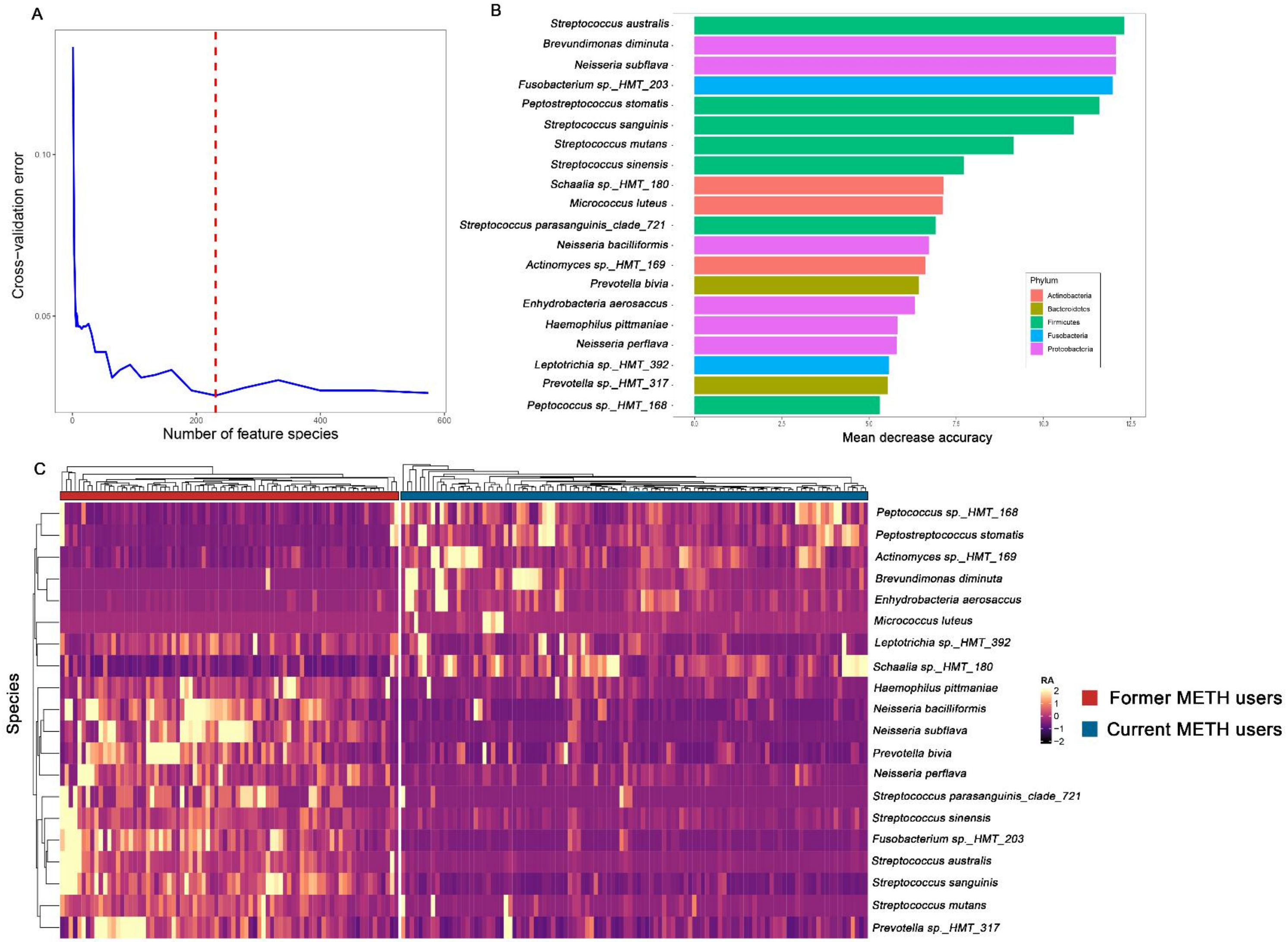

Fig. S10. Oral bacterial taxonomic biomarkers to distinguish current METH users and former METH users at the level of species. The top 231 biomarker bacterial species were identified by applying Random Forest classifier based on minimum value of 10-fold cross-validation error (A). And the top 20 biomarker taxa are ranked in descending order of importance to the accuracy of the model (B). The heatmap (the values were Z-score transformed) shows that the relative abundances of the top 20 biomarker taxa in the optimal markers when comparing current METH users with former METH users (C).

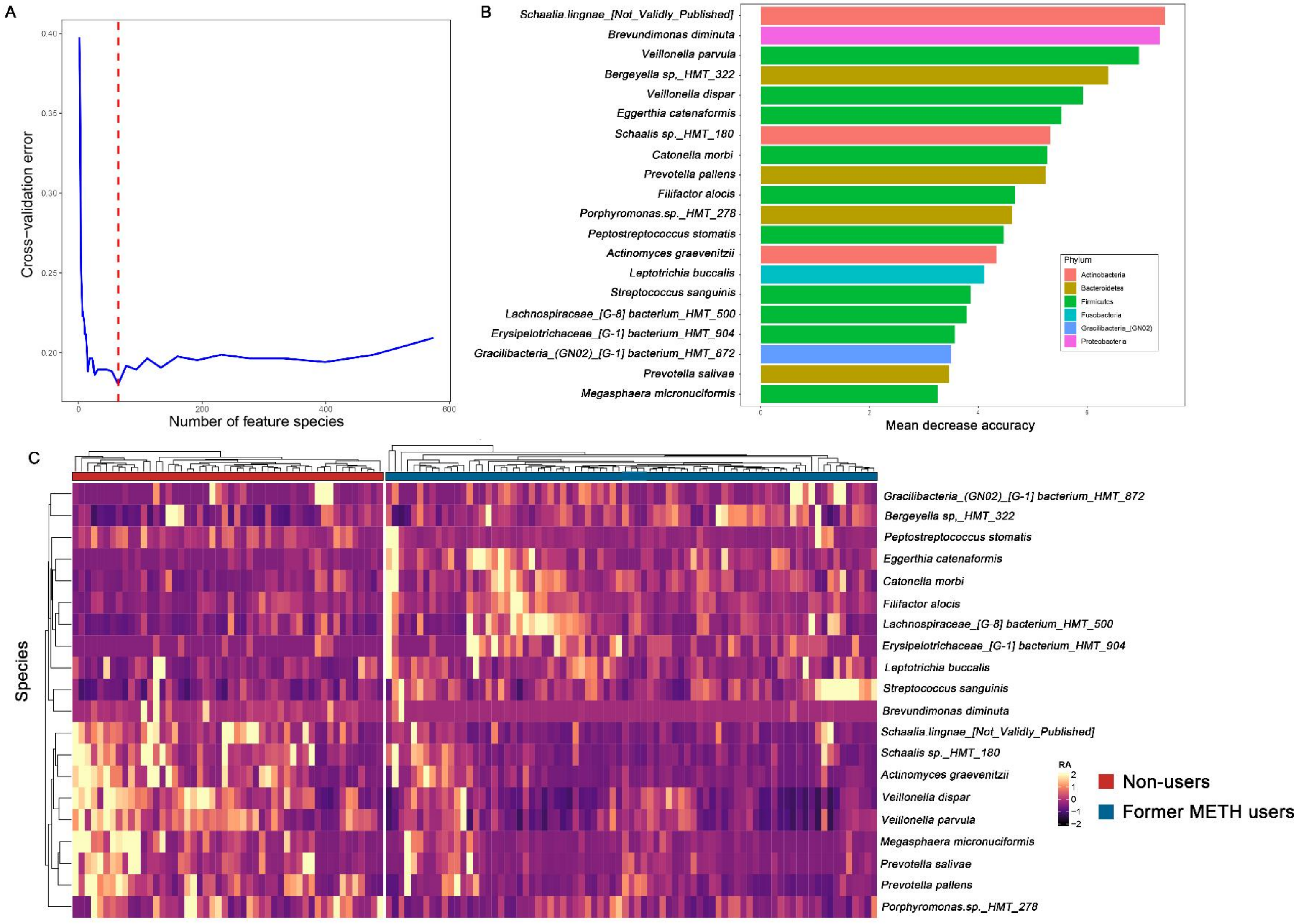

Fig. S11. Oral bacterial taxonomic biomarkers to distinguish former METH users and non-users at the level of species. The top 64 biomarker bacterial species were identified by applying Random Forest classifier based on minimum value of 10-fold cross-validation error (A). And the top 20 biomarker taxa are ranked in descending order of importance to the accuracy of the model (B). The heatmap (the values were Z-score transformed) shows that the relative abundances of the top 20 biomarker taxa in the optimal markers when comparing former METH users with non-users (C).

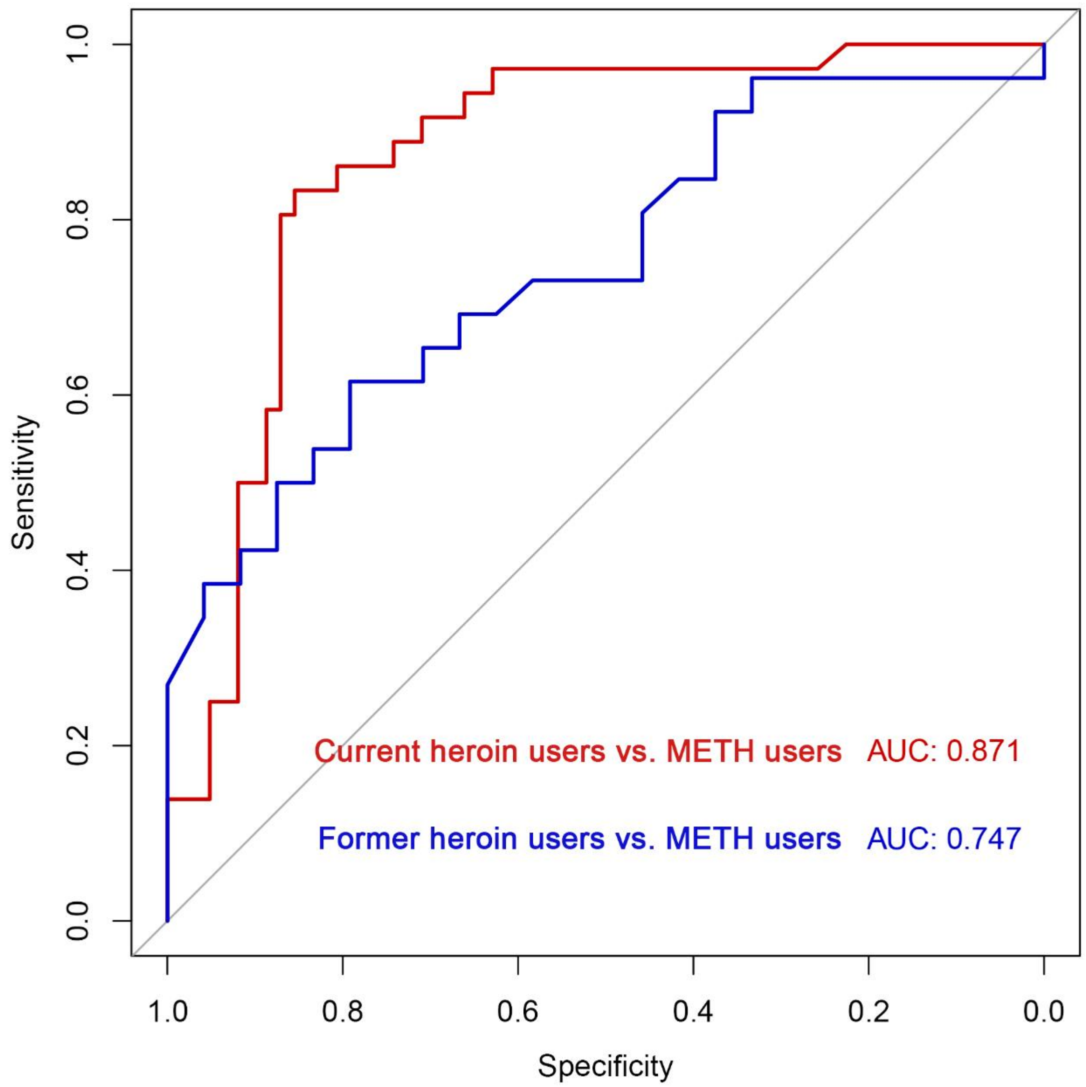

Fig. S12. ROC curves depicting classification performance of current heroin users versus current METH users (red line) and former heroin users versus former METH users (blue line) using the relative abundances of species based on random forest models.

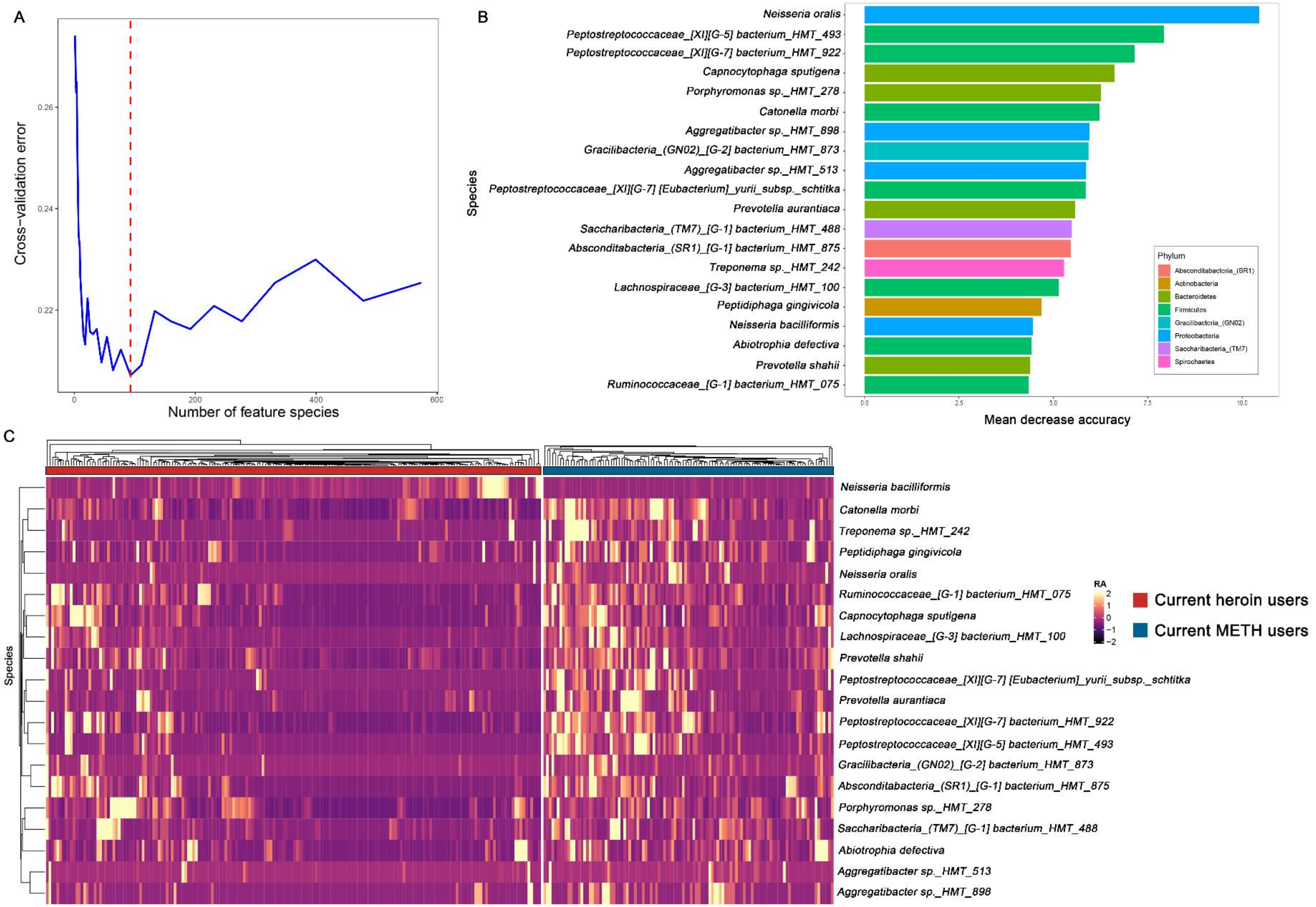

Fig. S13. Oral bacterial taxonomic biomarkers to distinguish current heroin users and current METH users at the level of species. The top 93 biomarker bacterial species were identified by applying Random Forest classifier based on minimum value of 10-fold cross-validation error (A). And biomarker taxa are ranked in descending order of importance to the accuracy of the model (B). The heatmap (the values were Z-score transformed) shows that the relative abundances of the top 20 biomarker taxa in the optimal markers when comparing current heroin users with current METH users (C).

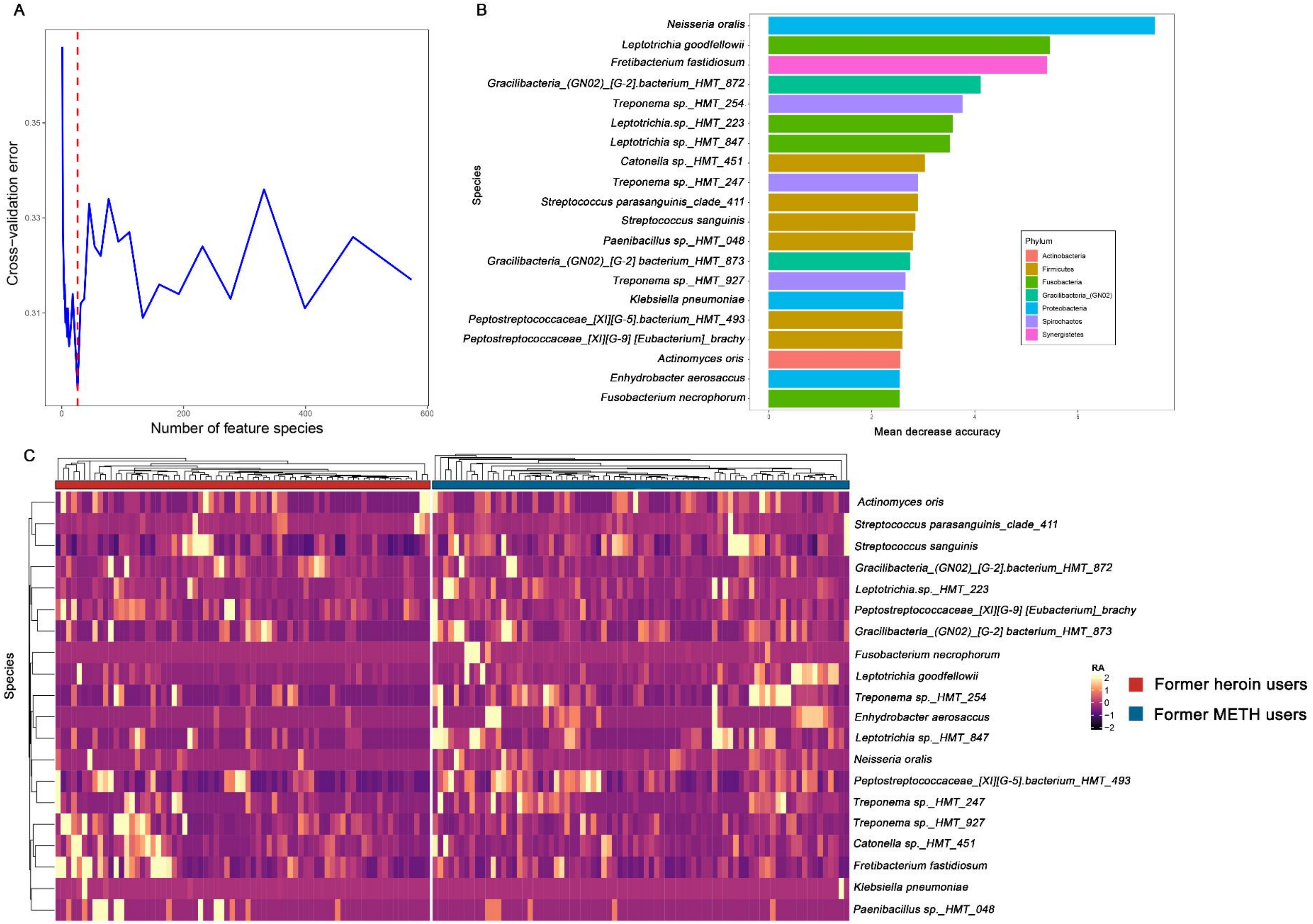

Fig. S14. Oral bacterial taxonomic biomarkers to distinguish former heroin users and former METH users at the level of species. The top 26 biomarker bacterial species were identified by applying Random Forest classifier based on minimum value of 10-fold cross-validation error (A). And the top 20 biomarker taxa are ranked in descending order of importance to the accuracy of the model (B). The heatmap (the values were Z-score transformed) shows that the relative abundances of the top 20 biomarker taxa in the optimal markers when comparing former heroin users with former METH users (C).
